# Effectiveness of bowel preparation innovative technology instructions (BPITIs) on clinical outcomes among patients undergoing colonoscopy: a systematic review and meta-analysis

**DOI:** 10.1038/s41598-023-37044-w

**Published:** 2023-07-04

**Authors:** Parichat Wonggom, Siwanon Rattanakanokchai, Orathai Suebkinorn

**Affiliations:** 1 Department of Adult Nursing, Faculty of Nursing, Khon Kaen University, Khon Kaen, 40002 Thailand; 2 Department of Epidemiology and Biostatistics, Faculty of Public Health, Khon Kaen University, Khon Kaen, 40002 Thailand

**Keywords:** Colonoscopy, Patient education

## Abstract

To evaluate the effectiveness of bowel preparation innovative technology instructions (BPITIs) among patients undergoing colonoscopy. We searched PubMed, MEDLINE, CINAHL, CENTRAL, Scopus, Web of Science, LILACS, ClinicalTrials.gov, and Google Scholar for randomised controlled trials (RCTs) and cluster-RCTs from inception to February 28, 2022. The Cochrane risk of bias (RoB) tool and GRADE were used to assess RoB and certainty of evidence, respectively. Meta-analyses with random-effects model were used for analysis. This review included 47 RCTs (84 records). Seven BPITIs were found among included studies: (1) mobile apps, (2) VDO stream from personal devices, (3) VDO stream from a hospital device, (4) SMS re-education, (5) telephone re-education, (6) computer-based education, and (7) web-based education. The findings demonstrate that BPITIs have a slight impact on adherence to overall instructions (RR 1.20, 95% CI 1.13–1.28; moderate-certainty evidence), adequate bowel preparation (RR 1.10, 95% CI 1.07–1.13; low-certainty evidence), and quality of bowel preparation score (SMD 0.42, 95% CI 0.33–0.52; low-certainty evidence) compared to routine care. BPITIs may enhance the clinical outcomes. Due to the low-certainty evidence and heterogeneity of the included studies, the findings should be interpreted cautiously. Well-designed and reported RCTs are required to confirm the findings.

*PROSPERO registration number*: CRD42021217846.

## Introduction

Colorectal cancer (CRC) is a major global health problem. According to the International Agency for Research on Cancer, CRC accounts for approximately 1.9 million new cases and more than 900,000 deaths each year^1^. CRC ranks as the third most cancer, next to breast and lung cancer, and the second leading cause of cancer-related deaths^2,3^. In addition, the incidence of CRC tends to increase in adults under the age of 50, which is defined as an early-onset CRC (EOCRC)^4^. Several studies have demonstrated that colonoscopy screening can prevent CRC incidence and mortality by detecting and removing precancerous lesions^5,6^. Therefore, a high-quality colonoscopy is required to achieve the desired screening benefit.

Adequate bowel preparation is essential for the success of a colonoscopy to visualize the colonic mucosa^6,7^. However, several studies indicate inadequate bowel preparations occurred in approximately 18–35%^8^. Inadequate bowel preparations lead to unsuccessful colonoscopy and negative consequences, such as prolonged colonoscopy time, decreased cecal intubation rates, decreased pathological lesions detection rates, required early repeat colonoscopy, and increased overall treatment costs^5,9^. Certain factors associated with inadequate bowel preparation include advanced age, male gender, inpatient status, chronic diseases (e.g., diabetes mellitus, hypertension, cirrhosis, and stroke), constipation, and narcotic and tricyclic antidepressant use^8^. Furthermore, a previous history of bowel preparation failure and non-adherence to bowel preparation instructions lead to sub-optimal bowel preparation^10^.

Numerous factors are associated with adequate bowel preparation, particularly those relating to healthcare providers and patients^11^. Healthcare providers are responsible for effectively educating patients on the bowel preparation process and emphasizing the significance of adequate bowel preparation. Patients undergoing colonoscopy must adhere to bowel preparation instructions, including dietary restrictions and proper purgative administration. Bowel preparation processes are complex and challenging for patients, particularly those undergoing their first colonoscopy^11^. Traditionally, healthcare professionals provide bowel preparation information with verbal or written instructions prior to colonoscopy day. These traditional education methods are unlikely enough to enable patients to comply with the bowel preparation instructions accurately, resulting in poor bowel cleansing, which negatively impacts the colonoscopy^12^. Therefore, strategies to optimize bowel preparation are critical.

Various innovative technology platforms have been used to enhance the quality of bowel preparation, such as smartphone-based, computer-based, and virtual reality videos. These technological platforms provide comprehensive instructions for bowel preparation in a timely manner, with a design that is both accessible and attractive. In addition, some technological platforms have a reminder function that assists patients in precisely following instructions and a communication function that enables patients to communicate directly with healthcare providers if they require additional information. Evidence revealed that these pre-colonoscopy educational programs could improve bowel cleanliness, adenoma and polyp detection rates, and adherence to bowel preparation instructions^12,13^. Therefore, innovative technology platforms have seemed to be widely utilised in bowel preparation before colonoscopy^14^.

Two systematic reviews were conducted to assess the effects of innovative technology education platforms on the quality of bowel preparation^14,15^. However, only the effects of mobile health (mHealth) technologies on colonoscopy preparation outcomes were evaluated in one review. Other innovative technology platforms, such as computer-based, virtual reality videos, and social media platforms, appear to improve bowel cleanliness^13,16,17^. Moreover, the findings of these two reviews were based on trials published prior to 2020. In recent years, further trials evaluating the effects of innovative technology instructions for bowel preparation have been published. We, therefore, conducted this systematic review to update the evidence regarding the effectiveness of bowel preparation innovative technology instructions (BPITIs) among patients undergoing colonoscopy by evaluating all available BPITIs.

## Methods

We conducted this systematic review following the Cochrane Handbook for Systematic Reviews of Interventions^18^. This systematic review was reported in accordance with the reporting guidance provided in the Preferred Reporting Items for Systematic Reviews and Meta-Analyses 2020 (PRISMA 2020) statement (Supplementary S1)^19^. The protocol of this systematic review can be found in the Prospective Register of Systematic Reviews (PROSPERO) (https://www.crd.york.ac.uk/prospero) with the registration number CRD42021217846.

### Eligibility criteria

We included individual randomised controlled trials (RCTs) and cluster randomised controlled trials (cluster RCTs) evaluating the effects of bowel preparation innovative technology instructions (BPITIs) in this review regardless of the language of publication, publication status, or year of publication. We included trials that included males and females aged 18 years and over undergoing colonoscopy. The BPITIs can be any bowel preparation interventions developed by using innovative technology to deliver bowel preparation information for patients to prepare their bowel for colonoscopy, such as mobile applications, telehealth, virtual social networks, or computer-based program. These interventions should be delivered to participants before they start their colonoscopy. Supplementary S2 outlines the definition of BPITIs that we found and grouped in our review. Comparators can be no intervention, usual care, or another bowel preparation innovative technology instruction(s).

### Types of outcome measures

Primary outcomes were adherence to instructions and bowel cleansing. Adherence to instructions can be assessed by adherence score (AS) and any validated instruments developed by a research team to use these instruments for their research. For bowel cleansing, this outcome should be assessed by the following measures: (1) the Aronchick scale, (2) the Ottawa Bowel Preparation Scale (OBPS), (3) the Boston Bowel Preparation Scale (BBPS), (4) the Chicago Bowel Preparation Scale, (5) the Harefield Cleansing Scale (HCS), (6) the Universal Preparation Assessment Scale (UPAS), and (7) any bowel preparation scales developed by a research team to use these scales for their research. The types of these two primary outcomes can be either dichotomous or continuous outcomes.

For secondary outcomes, we planned to evaluate the effects of BPITIs on polyp detection rates as assessed by the number of patients with polyps, and patient satisfaction as assessed by a numeric rating scale (NRS), a visual analogue scale (VAS), or a validated questionnaire which was developed by the research team who conducted that trial.

### Information sources

A systematic literature search was performed using the major electronic databases, including (1) PubMed, (2) MEDLINE (via Ovid), (3) CINAHL (via EBSCO), (4) Cochrane Central Register of Controlled Trials (CENTRAL), (5) Scopus, (6) ISI Web of Science, and (7) LILACS. We also reviewed reference lists of included trials and contacted trial authors to obtain additional data if necessary. In addition, we searched for ongoing studies in ClinicalTrials.gov, as well as unpublished studies in the Open Grey database. To ensure comprehensive searches, we searched for potential studies on Google Scholar, focusing on the first 50 records identified^20^.

### Search strategy

We systematically searched to identify all possible published and unpublished studies in the databases as mentioned above from their inception until February 28, 2022. The search strategy was developed using text words and medical subject headings (MeSH) according to the PICO model. Boolean operators including “AND” and “OR” were used to combine search terms for each PICO model or across PICO models, which were “colonoscope”, “bowel preparation”, “educational technologies”, and “randomised controlled trial”. Supplementary S3 presents the complete search strategy for each database.

### Selection process

**Figure 1:**
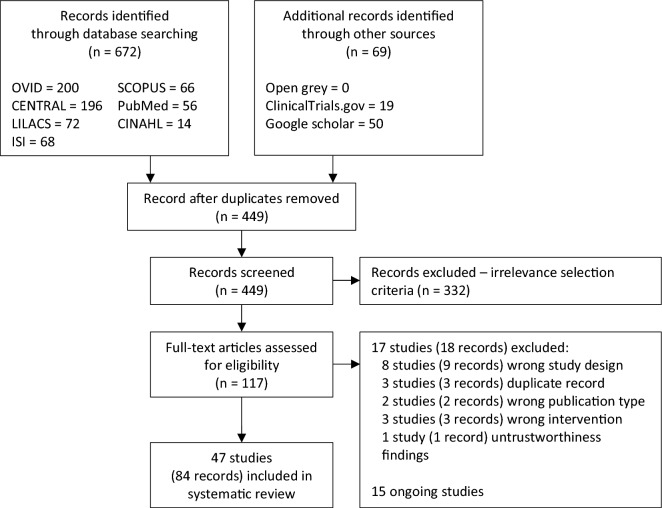
PRISMA diagram.

All records retrieved from each database were imported into Mendeley software to remove duplicate records. After duplicate records were removed, we then uploaded the records into Rayyan software. This online platform allows systematic reviewers to screen studies independently for titles and abstracts and full-text screening. At least two reviewers (PW, OS, and SR) independently screened titles and abstracts and full texts. Any disagreements between the two reviewers, either at the titles and abstracts screening stage or the full texts screening stage, were resolved by discussion; if required, the third reviewer was asked to reach a consensus. Studies published in a language other than Thai and English were translated into English using Google Translate. For any unclear data, the first or corresponding authors of the original study were contacted and requested to confirm unclear data. We presented the results of the search and selection process in the PRISMA flow diagram (Fig. 1).

### Data collection process

One of the review authors (SR) developed a data extraction form in Microsoft Access designed explicitly for this review. The form was piloted by all review authors before being implemented. At least two reviewers (PW, OS, and SR) independently extracted information from eligible trials using the agreed data extraction form. One of the reviewers (SR) checked for differences in the short text field data, drop-down list data, and numeric data extracted from two review authors using STATA software version 15.1. Any disagreements between two reviewers were resolved by discussion; if required, the third reviewer was asked to reach a consensus. We contacted the authors of the original study to request for confirm unclear or additional information. Plot digitizer software was used to extract information from graphs if the eligible trials did not provide numeric information for us to perform a meta-analysis in their report.

### Study risk of bias assessment

The Cochrane risk-of-bias tool version 1.0 for randomised controlled trials was used to assess the risk of bias among the eligible trials^21^. At least two reviewers (PW, OS, and SR) independently evaluated the risk of bias. Each included trial was assessed the risk of bias based on the following domains: (1) random sequence generation, (2) allocation concealment, (3) blinding of participants and personnel, (4) blinding of outcome assessment, (5) incomplete outcome data, and (6) selective reporting. We justified each risk of bias domain as “low risk of bias”, “high risk of bias”, and “unclear risk of bias”. Due to the nature of the trials evaluating the effects of an educational intervention for bowel preparation, participants and personnel could not be blinded. Therefore, all included studies were rated as having a high risk of bias for this domain, and this domain was not used to calculate the overall risk of bias for included studies. For the overall risk of bias, included studies were classified as high risk of bias if at least one RoB domain was classified as high risk of bias, as unclear risk of bias if at least one RoB domain was classified as unclear risk of bias but not at high risk of bias for any domain, and as low risk of bias if all RoB domains were classified as low risk of bias. Any discrepancies between the two reviewers were addressed through discussion, and if necessary, a third reviewer was invited to reach a conclusion.

### Effect measures

Relative risk (RR) was computed and presented for dichotomous outcomes, including adherence to instructions (overall, purgative intake, and dietary restrictions), adequate bowel preparation, polyp detection, and patient satisfaction. For continuous outcomes, mean difference (MD) and standardised mean difference (SMD) were computed and presented for outcomes that were measured using the same unit of measurement across trials and using different measurements across trials, respectively. The outcomes that were measured using different measurements included adherence to instructions (overall, purgative intake, and dietary restrictions), bowel preparation quality score, and satisfaction score. To interpret the SMD results, we followed Cohen’s effect sizes guideline outlined in the Cochrane handbook^22^. The following details are the SMD interpretation meaning that we used: (1) SMD less than 0.40 = small effect, (2) SMD greater than 0.40 and less than 0.69 = medium effect, and (3) SMD 0.70 or greater = large effect. Adjusted Odds Ratio (OR_adj_) and adjusted coefficient (Coef_adj_) were estimated and presented for adequate bowel preparation and bowel preparation quality score outcomes if the included trials provided effect estimates that were adjusted for confounding factors by statistical modelling. Separate analyses and presentations were made for both unadjusted and adjusted effect estimates.

We found that some of the included trials used the Ottawa Bowel Preparation Scale (OBPS) and the Aronchick Scale (AC) to measure bowel preparation quality. The directional scale of these two measurement tools differs from the other scales; a lower score means adequate bowel preparation for OBPS and AC, while a higher score means adequate bowel preparation for the other standardised bowel preparation scales. To make every tool have the same directional scale, we multiplied the mean values of trials that used (1) OBPS, (2) AC, and (3) bowel preparation scales that a lower score means adequate bowel preparation by − 1^23^.

### Synthesis methods

Study characteristics were presented in tabular format. We performed pair-wise meta-analyses using the random-effects model with the inverse variance weighting approach for all primary and secondary outcomes with at least two available trials^24^. We performed analyses on an intention‐to‐treat basis as far as possible. The results of random-effects models were presented in forest plots as the average treatment effects with 95% confidence intervals (CIs). RevMan software version 5.4.1 was used to perform meta-analysis for all analyses^25^.

For trials reporting zero events in one intervention arm, we added a correction factor of 0.5 into all cells of the study results to estimate the effect sizes. Trials with zero events in all intervention arms were removed from the meta-analysis. For trials that did not provide standard errors (SEs), we calculated SEs based on the width of CIs. If an included study reported treatment effects as a median and range or interquartile range, we computed the mean and standard deviation (SD) from these values using methods described by Wan and colleagues^26^.

We found trials with multiple intervention groups. To avoid double counting participants in the shared group (aka control group) and to address a correlation between the estimated intervention effects from multiple comparisons, we split the shared group into two or more groups depending on the number of intervention groups and adjusted its sample size by the number of comparisons. We then included two or more comparisons in meta-analyses^27^. For dichotomous outcomes, the number of events and the total number of participants were adjusted. For continuous outcomes, only the total number of participants was adjusted, while the means and standard deviations were unchanged.

We investigated statistical heterogeneity among trials included in each meta-analysis using visual inspection and statistical approaches. For the visual inspection approach, we considered the poor overlap of 95% CIs of the results of included trials in a forest plot. Statistical heterogeneity was identified if the I^2^ statistic value was greater than 75% and the *p* value from the Cochrane Q test was less than 0.10.

### Subgroup analysis and sensitivity analysis

We explored potential sources of heterogeneity for all primary outcomes by performing a subgroup analysis. Subgroup analyses were performed according to the following potential effect modifiers: (1) types of intervention and (2) types of bowel preparation scale. Sensitivity analyses were performed to assess the robustness of the findings by removing trials with the following factors: (1) unclear or high risk of overall risk of bias and (2) abstract only available.

### Publication bias assessment

A funnel plot was generated to assess publication bias for all primary outcomes if ten or more studies were included in a meta-analysis.

### Certainty of evidence

**Figure 2:**
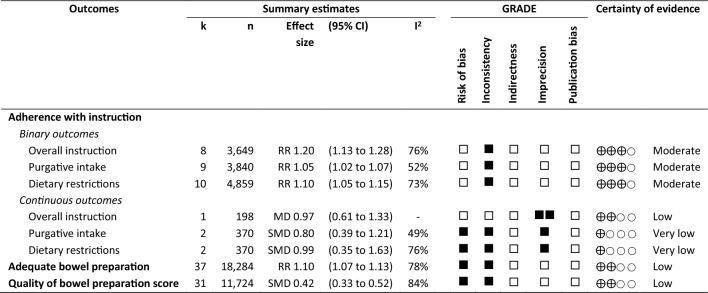
Summary of findings table for primary outcomes. ■, downgrade one level for each GRADE domain; ⨁, level of certainty of evidence; k, number of studies; n, number of participants; RR, risk ratio; MD, mean difference; SMD, standardized mean difference; CI, confidence interval.

The Grading of Recommendations, Assessment, Development and Evaluation (GRADE) approach was used to assess the certainty of the evidence for all primary outcomes across five domains: (1) risk of bias in the included studies, (2) inconsistency of results among included studies, (3) imprecision in the effect estimate, (4) indirectness of evidence, and (5) publication bias. The overall certainty of each piece of evidence was assessed as “very low”, “low”, “moderate”, or “high”. Two review authors (OS and SR) independently assessed the certainty of evidence; these results were then reviewed by the third review author (PW). All reviewers reached a consensus regarding the level of overall evidence certainty. GRADEPro GDT was utilised to construct the Summary of Findings (SoF) table^28^. Figure 2 illustrates a summary of findings table for primary outcomes. Supplemental S16 provides full details for the summary of findings table.

## Results

### Study selection

Figure 1 presents the PRISMA flow diagram for study selection. A systematic literature search identified 741 records from the major electronic databases, ClinicalTrials.gov, and Google Scholar. After removing 292 duplicate records, 449 records were independently screened by reviewing their titles and abstracts. 332 records were excluded because they did not meet the eligibility criteria, leaving 117 potential records for full-text reviews. 84 records of 47 studies met the eligibility criteria and were included in the quantitative synthesis^12,16,17,29–72^. (Supplementary S4) 18 records were excluded because of wrong study design (9 records), duplicate records (3 records), wrong publication type (2 records), wrong intervention (3 records), and untrustworthiness findings (1 record). Supplementary S5 shows the list of excluded studies with their exclusion reasons. 15 ongoing studies evaluating the effects of bowel preparation innovative technology instructions were identified. (Supplementary S6).

### Characteristics of include studies

Supplementary S7 summarises information on the characteristics of the included studies. Of the 47 included studies, all studies were individual randomised controlled trials. All trials were included in the quantitative analyses. Eight studies (17.0%) were reported in conference proceedings, with only abstracts being available^30,32,33,41,44,52,55,65^. Publication dates ranged from 2001 and 2022. We found six trials (10.4%) with multiple intervention groups; these trials were included in more than one comparison^39,41,45,51,56,69^. The included studies were conducted in 13 countries: the United States (12 studies)^32,36,40,49,50,55,57–59,62,64,67^, China (10 studies)^16,35,37,46,47,52,63,69,71,72^, the Republic of Korea (9 studies)^31,38,39,41,44,45,51,53,54^, Spain (three studies)^29,48,60^, Hong Kong^42,43^, Germany^12,68^, and the Netherlands^17,66^ (two studies each), and Australia^56^, Canada^65^, Denmark^34^, Lebanon^61^, Taiwan^70^, and the United Kingdom^30^ (one study each). One study did not report the study setting^33^ (Supplementary S8).

A variety of interventions were found in the included studies. To address this issue, we attempted to categorise the interventions into seven categories based on their main characteristics. Following are the seven types of interventions that we classified: 1) mobile apps, 2) VDO stream from personal devices, 3) short message services (SMS) re-education, 4) VDO stream from an on-site hospital device, 5) telephone re-education, 6) computer-based education, and 7) web-based education. The definition of each intervention can be found in Supplementary S2. All included studies compared these bowel preparation innovative technology instructions with routine care. The routine care consisted of bowel preparation instructions that were delivered by well-trained healthcare providers (physicians or nurses) with printed materials such as leaflets or booklets. Supplementary S9 presents contents of bowel preparation provided in each included study.

Among the 47 included studies, 45 studies (95.7%) reported at least one of the primary outcomes^12,16,17,29–55,57–61,63–72^. The two most common outcomes were adequate bowel preparation rate and quality of bowel preparation score outcomes, which were investigated in 37 trials (78.7%)^12,16,17,29,30,32,35–44,46–50,52–54,57,59–61,63–70,72^ and 34 trials (72.3%)^12,16,17,31–35,38–41,43–48,51–55,57–59,61,64,66–69,71,72^, respectively. (Supplementary S10) Various validated bowel preparation scales were used to evaluate the quality of bowel preparation in the included studies. We identified 26 included studies using the Boston Bowel Preparation Scale (BBPS)^12,17,29–33,35,37,39–41,44,45,55,59,60,63–69,71,72^, 10 using the Ottawa Bowel Preparation Scale (OBPS)^16,38,46,47,51–54,57,58^, five using the Aronchick scale (AC)^36,42,49,61,70^, one using the Harefield Cleansing Scale (HCS)^48^, and one using the AC and BBPS as measuring scales^43^. Two studies did not report the name of the bowel preparation scale used to evaluate bowel preparation quality in their trial report^34,50^. These 45 included studies quantified and analysed the quality of bowel preparation as either binary outcome (adequate bowel preparation rate) or continuous outcome (quality of bowel preparation score).

The included studies reported the following three outcomes for adherence to instructions: (1) adherence to overall instruction, (2) adherence to purgative intake, and (3) adherence to dietary restrictions. These three adherence outcomes were quantified and analysed as either binary or continuous outcomes in the included studies. Among these trials, the authors of the original study developed and validated their own measurement scale to quantify the adherence to instruction outcomes among their participants.

### Risk of bias in studies

**Figure 3:**
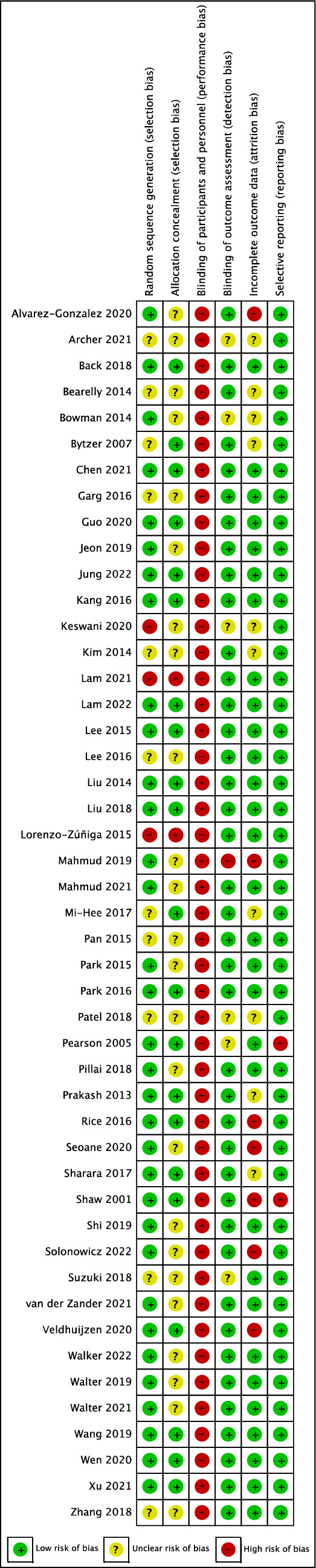
Summary risk of bias of included studies.

A summary risk of bias for each included study is presented in Fig. 3. 23 studies (48.9%) were classified as having an unclear risk of bias for the overall risk of bias^12,30,32–34,36,38,41,44,49,51,52,54,55,57,58,61,63,65–68,72^, 11 studies (23.4%) as having a high risk of bias^17,29,40,42,48,50,56,59,60,62,64^, and 13 studies (27.7%) as having low risk of bias^16,31,35,37,39,43,45–47,53,69–71^.

## Results of syntheses

### Primary outcomes

The primary outcomes were adherence to instructions and bowel preparation. The included studies reported three categories of adherence to instructions outcomes: 1) adherence to overall instruction, 2) adherence to purgative intake, and 3) adherence to dietary restrictions. The bowel preparation outcomes were presented as the rate of adequate bowel preparation and the quality of bowel preparation score as the following information.

### Adherence to instructions

**Figure 4:**
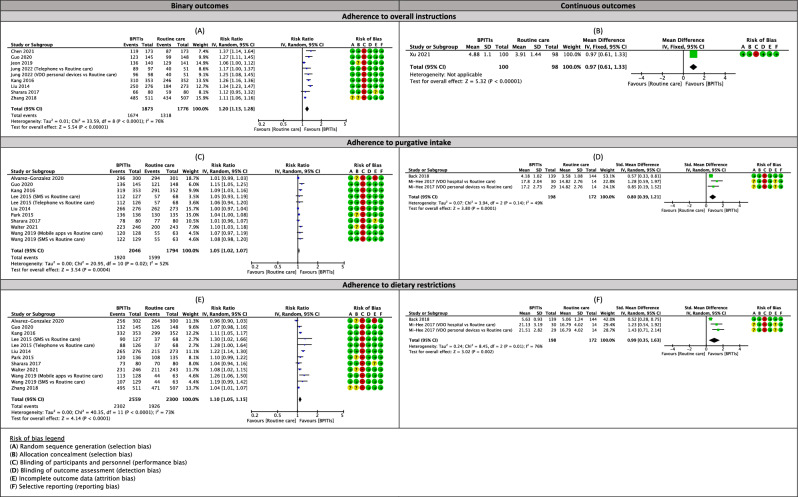
Effects of bowel preparation innovative technology interventions (BPITIs) on adherence with instructions for overall preparation, purgative intake, and diets. Telephone, telephone re-education; SMS, short message service; VDO hospital, video stream from an on-site hospital device; VDO personal devices, video stream from personal devices.

The meta-analyses and subgroup analyses by types of intervention for adherence to instructions outcomes are shown in Fig. 4, Table 1, and Supplementary S11.

**Table 1:** Subgroup analyses of the effects of bowel preparation innovative technology interventions (BPITIs) on all primary outcomes when compared with routine care by types of bowel preparation intervention.

Subgroups	Adherence to						Adequate bowel preparation	Quality of bowel preparation score
	Binary outcomes			Continuous outcomes				
	Overall instruction	Purgative intake	Dietary restrictions	Overall instruction	Purgative intake	Dietary restrictions		
	RR (95% CI)	RR (95% CI)	RR (95% CI)	MD (95% CI)	SMD (95% CI)	SMD (95% CI)	RR (95% CI)	SMD (95% CI)
Whole sample	**1.20** **(1.13 to 1.28)**	**1.05** **(1.02 to 1.07)**	**1.10** **(1.05 to 1.15)**	**0.97** **(0.61 to 1.33)**	**0.80** **(0.39 to 1.21)**	**0.99** **(0.35 to 1.63)**	**1.10** **(1.07 to 1.13)**	**0.42** **(0.33 to 0.52)**
I^2^	76%	52%	73%	–	49%	76%	78%	84%
Studies	8	9	10	1	2	2	37	31
Participants	3649	3840	4859	198	370	370	18,284	11,724
Computer based education	No data	No data	No data	No data	No data	No data	1.01 (0.96 to 1.06)	**− **0.12 (**− **0.30 to 0.06)
I^2^							27%	–
Studies							2	1
Participants							1102	497
Mobile apps	**1.18** **(1.09 to 1.29)**	**1.08** **(1.03 to 1.13)**	**1.07** **(1.04 to 1.11)**	No data	No data	No data	**1.11** **(1.06 to 1.16)**	**0.43** **(0.30 to 0.57)**
I^2^	70%	45%	41%				74%	79%
Studies	4	5	6				12	10
Participants	2176	1838	2856				5394	4685
SMS	No data	**1.04** **(1.01 to 1.08)**	**1.14** **(1.05 to 1.24)**	No data	No data	No data	**1.08** **(1.03 to 1.13)**	**0.39** **(0.20 to 0.59)**
I^2^		0%	0%				68%	69%
Studies		3	3				9	6
Participants		658	658				5037	1499
Telephone	**1.27** **(1.11 to 1.45)**	1.01 (0.99 to 1.03)	1.13 (0.93 to 1.37)	No data	No data	No data	**1.10** **(1.03 to 1.18)**	**0.66** **(0.53 to 0.78)**
I^2^	56%	0%	93%				69%	0%
Studies	2	3	3				5	4
Participants	697	1344	1345				2445	1035
VDO hospital	**1.37** **(1.14 to 1.64)**	No data	No data	No data	**1.28** **(0.59 to 1.97)**	**1.23** **(0.54 to 1.92)**	**1.21** **(1.08 to 1.34)**	**0.41** **(− 0.08 to 0.91)**
I^2^	–				–	–	39%	92%
Studies	1				1	1	4	4
Participants	346				44	44	1020	1028
VDO personal devices	1.13 (0.97 to 1.33)	No data	No data	No data	**0.60** **(0.38 to 0.83)**	**0.91** **(0.03 to 1.79)**	**1.09** **(1.02 to 1.18)**	**0.42** **(0.23 to 0.61)**
I^2^	–				0%	82%	83%	83%
Studies	2				2	2	8	12
Participants	430				326	326	2836	3330
Web-based education	No data	No data	No data	No data	No data	No data	**1.27** **(1.02 to 1.58)**	No data
I^2^							–	
Studies							1	
Participants							188	

Significant values are in bold.

#### Adherence to overall instruction

Bowel preparation innovative technology instructions (BPITIs) probably increase the rate of adherence to overall instruction slightly when compared to routine care (RR 1.20, 95% CI 1.13 to 1.28; 8 studies; 3649 participants; moderate-certainty evidence; Fig. 4A)^16,35,37–39,47,61,72^, although there was substantial heterogeneity for this outcome (I^2^ = 76%; *p* value < 0.001). Only one study (198 participants) reported adherence score to overall instruction^71^; this trial evaluated the effect of VDO stream from personal devices on this outcome. The evidence suggests that this intervention results in a slight increase in the adherence score to overall instruction (MD 0.97, 95% CI 0.61 to 1.33; low-certainty evidence; Fig. 4B). Subgroup analysis indicated no significant difference in the adherence rate to overall instruction outcome across the types of intervention (*p* value 0.35). However, the evidence of this subgroup analysis is very uncertain about the effect of VDO stream from personal devices on this outcome (RR 1.13, 95% CI 0.97 to 1.13; 2 studies; 430 participants; Supplementary S11B)^38,39^.

#### Adherence to purgative intake

The evidence suggests BPITIs result in a slight increase in the rate of adherence to purgative intake when compared to routine care (RR 1.05, 95% CI 1.02 to 1.07; 9 studies; 3840 participants; moderate-certainty evidence; Fig. 4C)^12,16,29,37,45,47,54,61,69^, although there was moderate heterogeneity for this outcome (I^2^ = 52%; *p* value = 0.02). The same result was seen on the adherence score to purgative intake outcome, in which BPITIs may increase this outcome compared to routine care but the evidence is very uncertain (SMD 0.80, 95% CI 0.39 to 1.21; 2 studies; 370 participants; very low-certainty evidence; Fig. 4D)^31,51^. However, there was no serious heterogeneity for this outcome (I^2^ = 49%; *p* value = 0.14). Subgroup analysis indicated that the type of intervention might make a difference in the adherence rate to purgative intake (*p* value = 0.009). Mobile apps (RR 1.08, 95% CI 1.03 to 1.13; 5 studies; 1838 participants; Supplementary S11C)^12,16,37,61,69^ and SMS (RR 1.04, 95% CI 1.01 to 1.08; 3 studies; 658 participants; Supplementary S11C)^45,54,69^ may increase the rate of adherence to purgative intake, but telephone re-education has little to no effect on this outcome (RR 1.01; 95% CI 0.99 to 1.03; 3 studies; 1344 participants; Supplementary S11C)^29,45,47^.

#### Adherence to dietary restrictions

BPITIs are likely to result in a slight increase in the rate of adherence to dietary restrictions compared to routine care (RR 1.10, 95% CI 1.05 to 1.15; 10 studies; 4859 participants; moderate-certainty evidence; Fig. 4E)^12,16,29,37,45,47,54,61,69,72^. Substantial statistical heterogeneity was noted for this outcome (I^2^ = 73%; *p* value < 0.001). Two trials involving 370 participants reported the dietary restrictions adherence score. The result showed that BPITIs probably result in an increase in this outcome when compared to routine care but the evidence is very uncertain (SMD 0.99, 95% CI 0.35 to 1.63; 2 studies; 370 participants; very low-certainty evidence; Fig. 4F)^31,51^. The heterogeneity was not identified in this meta-analysis (I^2^ = 0%; *p* value = 0.57). Subgroup analysis by types of intervention could not explain the source of heterogeneity for the rate of adherence to dietary restrictions outcome (*p* value = 0.38).

#### Sensitivity analysis and publication bias assessment

Sensitivity analyses that eliminated the trials with an unclear or high risk of overall risk of bias and trials that abstract only available did not alter the results of these outcomes (Table 2). Only the meta-analysis of the rate of adherence to dietary restrictions outcome has a sufficient number of studies to investigate for publication bias. The funnel plot for this outcome was symmetrical (Supplementary S15A).

**Table 2:** Sensitivity analyses by excluding abstract-only RCTs and unclear or high risk of bias RCTs.

Sensitivity analyses	Adherence to						Adequate bowel preparation	Quality of bowel preparation score
	Binary outcomes			Continuous outcomes				
	Overall instruction	Purgative intake	Dietary restrictions	Overall instruction	Purgative intake	Dietary restrictions		
	RR (95% CI)	RR (95% CI)	RR (95% CI)	MD (95% CI)	SMD (95% CI)	SMD (95% CI)	RR (95% CI)	SMD (95% CI)
Whole sample	**1.20** **(1.13 to 1.28)**	**1.05** **(1.02 to 1.07)**	**1.10** **(1.05 to 1.15)**	**0.97** **(0.61 to 1.33)**	**0.80** **(0.39 to 1.21)**	**0.99 ** **(0.35 to 1.63)**	**1.10** **(1.07 to 1.13)**	**0.42** **(0.33 to 0.52)**
I^2^	76%	52%	73%	–	49%	76%	78%	84%
Studies	9	11	12	1	3	3	37	31
Participants	3649	3840	4859	198	370	370	18,284	11,724
Studies with full-text	**1.20 ** **(1.13 to 1.28)**	**1.05** **(1.02 to 1.07)**	**1.10 ** **(1.05 to 1.15)**	**0.97** **(0.61 to 1.33)**	**0.80** **(0.39 to 1.21)**	**0.99** **(0.35 to 1.63)**	**1.09 ** **(1.06 to 1.12)**	**0.43** **(0.33 to 0.54)**
I^2^	76%	52%	73%	–	49%	76%	78%	85%
Studies	9	11	12	1	3	3	32	26
Participants	3649	3840	4859	198	370	370	15,914	10,020
Studies with low RoB	**1.28** **(1.22 to 1.34)**	**1.07 ** **(1.02 to 1.11)**	**1.16** **(1.10 to 1.23)**	**0.97** **(0.61 to 1.33)**	**0.60** **(0.36 to 0.84)**	**0.57 ** **(0.32 to 0.82)**	**1.17** **(1.13 to 1.21)**	**0.57** **(0.45 to 0.70)**
I^2^	0%	54%	45%	–	–	–	21%	76%
Studies	5	5	5	1	1	1	11	11
Participants	2190	2319	2319	198	283	283	4892	4812

RR, risk ratio; MD, mean difference; SMD, standardized mean difference.

Significant values are in bold.

### Bowel preparation

**Figure 5:**
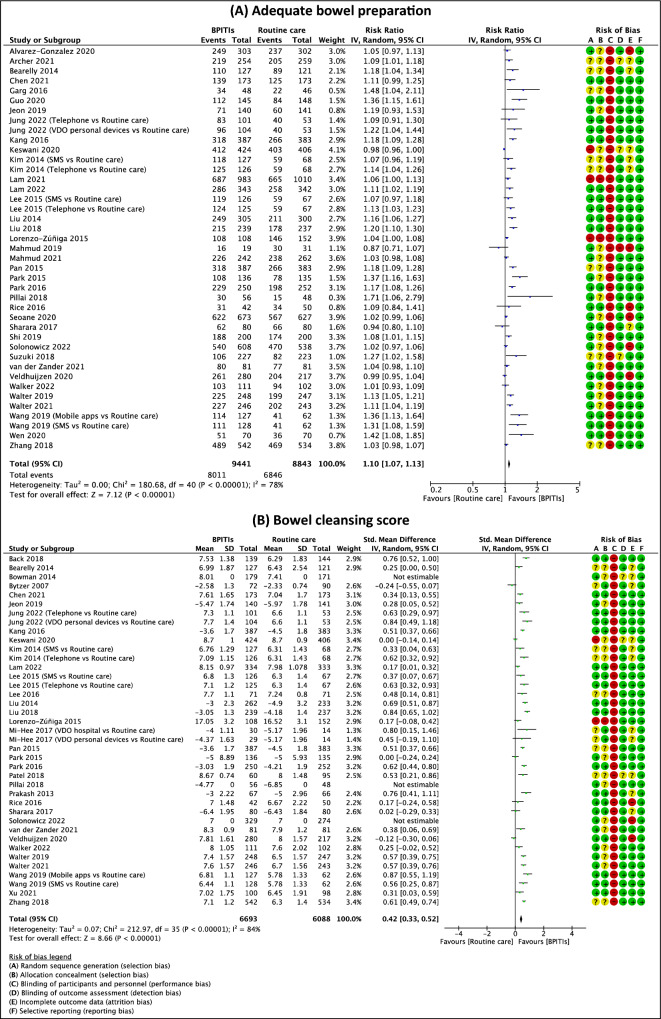
Effects of bowel preparation innovative technology interventions (BPITIs) on (**A**) adequate bowel preparation and (**B**) bowel cleansing score. Telephone, telephone re-education; SMS, short message service; VDO hospital, video stream from an on-site hospital device; VDO personal devices, video stream from personal devices.

**Figure 6:**
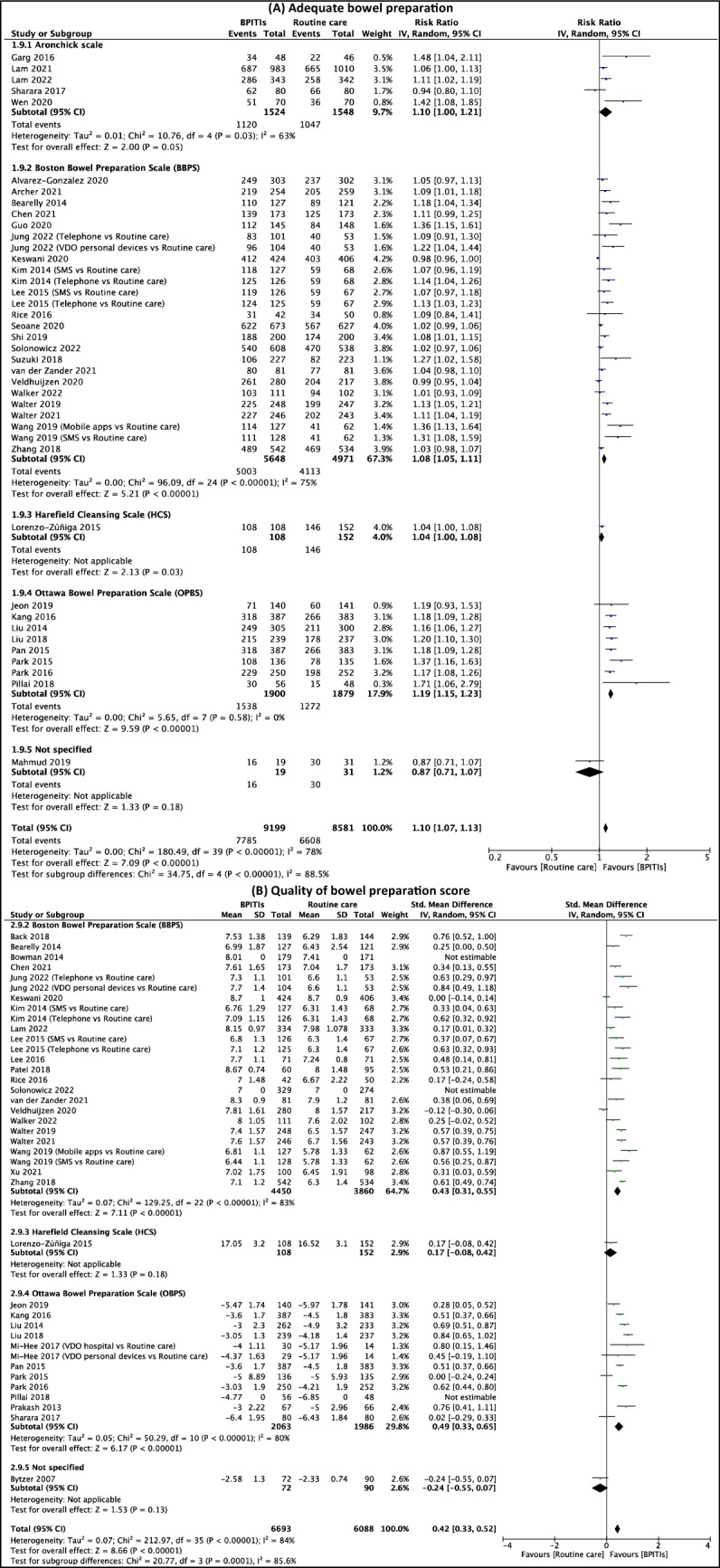
Subgroup analyses of the effects of bowel preparation innovative technology interventions (BPITIs) on (**A**) adequate bowel preparation and (**B**) bowel cleansing score by bowel preparation quality scales. Telephone, telephone re-education; SMS, short message service; VDO hospital, video stream from an on-site hospital device; VDO personal devices, video stream from personal devices.

Figure 5 presents the meta-analysis findings on (1) adequate bowel preparation rate and (2) quality of bowel preparation score. The findings of subgroup analyses by types of bowel preparation scale and types of intervention can also be found in Fig. 6, Table 1, and Supplementary S12-S13. Three included studies did not report the standard deviations (SDs) for both the intervention and comparison groups^33,58,64^. Therefore, these trials were not comprised in the quantitative analyses of the quality of bowel preparation score outcome.

#### Rate of adequate bowel preparation

The evidence suggests BPITIs result in a slight increase in the rate of adequate bowel preparation when compared to routine care (RR 1.10, 95% CI 1.07 to 1.13; 37 studies; 18,284 participants; low-certainty evidence; Fig. 5A)^12,16,17,29,30,32,35–43,45–50,52–54,57,59–61,63–70,72^, although there was substantial heterogeneity for this outcome (I^2^ = 78%; *p* value < 0.001). Subgroup analysis revealed that the types of bowel preparation scale and treatment may have impacted the pooled effect of this outcome. For types of intervention, most interventions may increase this outcome slightly, except for computer-based education which has little to no impact on this outcome, but the evidence is very uncertain (RR 1.01, 95% CI 0.96 to 1.06; 2 studies; 1102 participants; Supplementary S12A)^17,29^. The finding of subgroup analysis by bowel preparation scale indicated that trials that used BBPS (RR 1.08, 95% CI 1.05 to 1.11; 21 studies; 10,619 participants; Fig. 6A)^12,17,29,30,32,35,37,39–41,45,59,60,63–69,72^ and OBPS (RR 1.19, 95% CI 1.15 to 1.23; 8 studies; 3779 participants; Fig. 6A)^16,38,46,47,52–54,57^ might result in a better in the rate of adequate bowel preparation.

Five included studies reported the adjusted odds ratio (OR_adj_) for the adequate bowel preparation outcome^31,43,45,53,54^. The evidence suggests BPITIs result in a large increase in the rate of adequate bowel preparation (OR_adj_ 2.92, 95% CI 1.82 to 4.67; 5 studies; I^2^ = 60%) (Table 3 and Supplementary S14A).

**Table 3:** Meta analyses of the effects of bowel preparation innovative technology interventions (BPITIs) on primary outcomes adjusted for confounding factors and secondary outcomes when compared with routine care.

	Summary estimates*			No. of studies	No. of participants
	Effect size	(95% CI)	I^2^		
Primary outcomes					
Adequate bowel preparation	OR_adj_ 2.92	(1.82 to 4.67)	60%	5	–
Bowel cleansing score	Coef_adj_ 0.82	(0.50 to 1.14)	–	1	–
Secondary outcomes					
Polyp detection	RR 1.22	(1.10 to 1.35)	48%	20	7616
Satisfied with intervention	RR 1.38	(1.16 to 1.65)	71%	5	1746
Satisfaction score	SMD 0.62	(0.37 to 0.86)	87%	10	2557

*Inverse variance method with random effects model.

#### Quality of bowel preparation score

BPITIs may increase the quality of bowel preparation score slightly compared to routine care (SMD 0.42, 95% CI 0.33 to 0.52; 31 studies; 11,724 participants; low-certainty evidence; Fig. 5B)^12,16,17,31,32,34,35,38–41,43–48,51–55,58,59,61,66–69,71,72^. Substantial statistical heterogeneity was noted for this outcome (I^2^ = 84%; *p* value < 0.001). Subgroup analysis also indicated that the types of bowel preparation scale and treatment may have impacted the pooled effect of this outcome. For types of intervention, most interventions may improve this outcome slightly, except for computer-based education (SMD -0.12, 95% CI -0.30 to 0.06; 1 study; 497 participants; Supplementary S12B)^17^ and VDO stream from an on-site hospital device (SMD 0.41, 95% CI -0.08 to 0.91; 4 studies; 1028 participants; Supplementary S12B)^34,35,46,51^ which have little to no effect on this outcome, but the evidence is very uncertain. The finding of subgroup analysis by types of bowel preparation scale indicated that trials that used BBPS (MD 0.60, 95% CI 0.42 to 0.77; 19 studies; 7357 participants; Supplementary S13)^12,17,31,32,35,39–41,43–45,55,59,66–69,71,72^ and OBPS (MD -1.00, 95% CI -1.27 to -0.73; 10 studies; 3945 participants; Supplementary S13)^16,38,46,47,51–54,58,61^ might result in a better in the quality of bowel preparation score.

One trial comparing the effect of SMS and routine care on the quality of bowel preparation score reported the adjusted coefficient (coef_adj_) for this outcome^68^. The confounding factor that was adjusted in this trial was gender. The evidence suggests SMS might improve this outcome slightly (coef_adj_ 0.82, 95% CI 0.50 to 1.14; 1 study) (Table 3).

#### Sensitivity analysis and publication bias assessment

Sensitivity analyses revealed that the results of (1) adequate bowel preparation rate and (2) quality of bowel preparation score were not affected by removing the trials with an unclear or high risk of overall risk of bias and trials that abstract only available. (Table 2) The funnel plot for (1) the adequate bowel preparation rate and (2) the quality of bowel preparation score outcomes were somewhat asymmetrical, indicating some publication bias for these two outcomes (Supplementary S15B and S15C).

### Secondary outcomes

Secondary outcomes include polyp detection rate and satisfaction, presented in the following section.

### Polyp detection rate

The evidence suggests BPITIs increase polyp detection slightly when compared to routine care (RR 1.22, 95% CI 1.10 to 1.35; 20 studies; 7616 participants)^16,29,30,32,35–39,41,44–47,52–54,66,67,69^, although there was substantial heterogeneity for this outcome (I^2^ = 48%; *p* value = 0.004) (Table 3 and Supplementary S14B).

### Satisfaction

Two included studies only reported the rate of participants satisfied with intervention in the intervention group^12,50^. In addition, one trial did not report the SDs for the intervention and comparison groups for satisfaction score outcome^64^. Thus, these trials were not included in the quantitative analyses of (1) satisfaction with the intervention and (2) satisfaction score.

BPITIs may result in a slight increase in satisfaction with the intervention compared to routine care (RR 1.38, 95% CI 1.16 to 1.65; 5 studies; 1746 participants)^45,58,60,62,69^. Substantial statistical heterogeneity was noted for this outcome (I^2^ = 71%; *p* value < 0.001). The same result was seen in the satisfaction score outcome, in which BPITIs may increase this outcome compared to routine care (SMD 0.62, 95% CI 0.37 to 0.86; 10 studies; 2557 participants)^31,35,39,48,51,60–62,66,70^ (Table 3 and Supplementary S14C and S14D).

## Discussion

This systematic review aimed to investigate the effectiveness of bowel preparation innovative technology instructions (BPITIs) for patients undergoing colonoscopy on clinical outcomes, including adherence to instructions, quality of bowel preparation, polyp detection, and participants’ satisfaction. The findings of this systematic review indicate that the use of BPITIs for colonoscopy is likely associated with a slight improvement in all outcomes, as mentioned above. However, statistical heterogeneity was found to be moderate to high for all outcomes measured and the risk of publication bias was high. Different intervention types may have varying effects on adherence to purgative intake and the quality of bowel preparation. In addition, the quality of bowel preparation was also affected by the types of bowel preparation quality scale. Sensitivity analyses that excluded the trials with an unclear or high risk of overall risk of bias and trials with only an abstract available revealed no marked differences in the direction and magnitude of these associations.

We found two recent systematic reviews evaluating the effects of innovative technologies supporting colonoscopy preparation on patient and clinical outcomes; one review using traditional pair-wise meta-analysis^14^ and the other using network meta-analysis^15^.

Bizri et al., 2021^14^ compared Mobile health technologies (mHealth) with usual care. This review included 10 RCTs; all 10 studies were included in our review. They found that mHealth technologies are likely to improve the quality of bowel preparation which was similar to one of our subgroup analysis findings which observed the effects of mobile applications.

Tian et al., 2021^15^ performed a systematic review and network meta-analysis on bowel preparation instructions to deliver bowel preparation information for patients before colonoscopy. This review included 23 RCTs with 10 different bowel preparation technology instructions, including SMS text messaging, telephone calls, newly designed booklets, new visual aids, mobile apps, educational videos, additional explanations, visual aids, and social media applications. Due to the abilities of network meta-analysis, this review is able to compare more than two interventions in a single analysis, determine indirect effects for interventions that have not been directly compared, combine direct and indirect effects which can yield more precise evidence when both direct and indirect effects are available, and rank interventions. In conclusion, they concluded that newly designed booklets, telephone calls, educational videos, and social media applications probably increase the rate of adequate bowel preparation. In addition, telephone calls and social media applications may promote adherence to bowel preparation instructions, reduce the risk of adverse events, and increase the polyp detection rate. These findings are in line with our review.

This review included 47 RCTs. The study participants were recruited from 13 countries, mainly from the United States, China, and the Republic of Korea. Only one study was undertaken in a lower-middle-income country, while 35 were conducted in high-income countries. Therefore, healthcare providers and policymakers working in lower-middle-income countries should interpret the findings of this review with caution, considering the potential limitations in terms of resources and accessibility to technology^73,74^. The included studies were published between 2001 to 2022; three RCTs were published prior to 2010, 33 RCTs were published between 2011 to 2020, and 11 RCTs were published after 2020. It is possible that some BPITIs may not be generalisable to the current clinical context. All participants in the included RCTs that reported an indication for colonoscopy were performed colonoscopy for investigation. The study participants were adults or elderly. One trial exclusively included participants who had never undergone a colonoscopy before, but the rest of the trials included participants with or without previous colonoscopy experience.

The risk of bias varied across the included studies. Two-thirds of included studies lacked detailed descriptions of methods for random sequence generation and allocation concealment. Consequently, a substantial proportion of the trials were classified as having unclear risk of bias for the overall risk of bias. Three studies were deemed inadequate random sequence generation because participants were randomly assigned by odd or even colonoscopy date, odd or even medical record number, and the type of participant’s smartphone (iOS vs others). Due to the nature of the intervention, where participants and personnel could not be blinded, we classed all trials as having a high risk of performance bias. In this systematic review, the certainty of evidence ranges from very low to moderate. Most of the evidence was downgraded due to the heterogeneity of the included trials. Other factors for downgrading included a lack of clarity or a high risk of bias in the included studies, as well as imprecision (small sample sizes and the limited number of available studies). Potential publication bias was revealed in two of three primary outcomes with ten or more included studies: 1) rate of adherence to dietary restrictions outcome and 2) rate of adequate bowel preparation. We identified 15 trial registrations. The registration dates for these trial registrations range from 2017 to 2021. Uncertainty regarding the status of some trial registrations, whether recruitment is ongoing or complete. As a result, there is a potential risk, as the outcomes of these trials have not been published. The results of this systematic review must therefore be regarded with caution in light of these challenges.

To the best of our knowledge, this is the largest systematic review of bowel preparation innovative technology instructions for colonoscopy. We are aware that bias could occur at any stage during the reviewing process. As a result, we attempt to reduce bias using various methods. A systematic literature search was performed to retrieve all possible RCTs and cluster RCTs in several major electronic databases, as well as the reference list of included trials, regardless of publication status, publication date, and language. Authors of the original study were contacted to confirm unclear information or request additional information. Two review authors independently screened studies, extracted data, assessed the risk of bias, and assessed the certainty of evidence. To minimise errors that may arise during the data extraction process, we created an electronic data extraction form designed explicitly for this review in Microsoft Access, a database management platform. Any differences between the two reviewers in these processes were addressed through discussion, and if necessary, a third reviewer was requested to reach a conclusion. Furthermore, our review determined and presented in greater detail about the characteristics of the included studies, study setting, reported outcomes, and contents of bowel preparation provided in each included study.

However, some obstacles may have had an impact on our results. Due to a variety of bowel preparation innovative technology interventions found in this review, we categorised the interventions into seven categories based on their main characteristics to reduce the number of possible comparisons. There is currently no standard definition for grouping these interventions. This approach may introduce bias into our results. We attempted to make this issue as transparent as possible by providing the description we used in supplemental S2. The range of bowel preparation contents provided in each included study can also lead to varying findings. However, subgroup or meta-regression analyses were not conducted to investigate this factor. Due to the lack of a clinical guideline that specifies the minimal contents that should be provided to patients undergoing colonoscopy, we cannot classify interventions based on this factor. Publication bias, a high risk of selection bias due to the nature of this intervention in which participants and health personnel could not be blinded, and substantial heterogeneity identified in almost all meta-analyses are other biases that should be taken into account in this study. One of the sources of heterogeneity was types of bowel preparation quality scale. The included studies used the following four validated bowel preparation scales to evaluate the quality of bowel preparation: (1) the Boston Bowel Preparation Scale (BBPS), (2) the Ottawa Bowel Preparation Scale (OBPS), (3) the Aronchick scale (AC), and (4) the Harefield Cleansing Scale (HCS). Thus, the gold standard bowel preparation and adherence to instruction tools should be developed for reliable measurement.

In a real-world clinical setting, some BPITIs may not be suitable for all participants. Those with low digital health literacy and cognitive impairment, for instance, may be less likely to benefit from advanced technology platforms. Therefore, once developing and implementing innovative technology platforms to enhance the quality of bowel preparation, it is necessary to consider the characteristic of participants, particularly their needs, preferences, and ability to access innovative technology platforms. In addition, well-designed RCTs using validated tools to evaluate outcomes are still required to reaffirm our findings, and the results should be strictly reported by following the CONSORT guidelines. Future studies might determine the effects of innovative interventions regarding bowel preparation to reduce patients' pre-procedural anxiety and increase user satisfaction. We did not find studies examining the cost-effectiveness of these technologies. Therefore, further studies regarding the cost-effectiveness of these platforms might be required.

## Conclusion

This meta-analysis provides substantial evidence that bowel preparation innovative technology instructions may slightly improve overall adherence to instructions, adequate bowel preparation, and quality of bowel preparation score. The findings should be interpreted with caution due to the low and moderate certainty of evidence and heterogeneity among the included studies since the types of intervention and types of bowel preparation quality scale.

## Supplementary Information

Supplementary Information.

## Data Availability

All data generated or analysed during this study are included in this published article or the supplementary file.
