# Supplementary material for: Effectiveness of bowel preparation innovative technology instructions (BPITIs) on clinical outcomes among patients undergoing colonoscopy: a systematic review and meta-analysis
Source: Sci Rep. 2023 Jul 4;13:10783. doi: 10.1038/s41598-023-37044-w (PMC10319802; doi:10.1038/s41598-023-37044-w)
Supplement: Supplementary file 1 — Supplementary Information. [file 41598_2023_37044_MOESM1_ESM.pdf]

Supplementary information

Title: Effectiveness of bowel preparation innovative technology instructions (BPITIs) on clinical outcomes among patients undergoing colonoscopy: a systematic review and meta-analysis

Authors:

Parichat Wonggom [1], Siwanon Rattanakanokchai [2], Orathai Suebkinorn [1*]

Affiliations:

[1] Department of Adult Nursing, Faculty of Nursing, Khon Kaen University, Khon Kaen, Thailand, 40002

[2] Department of Epidemiology and Biostatistics, Faculty of Public Health, Khon Kaen University, Khon Kaen, Thailand, 40002

***Corresponding author:**

Name: Orathai Suebkinorn

Address: Lecturer, Department of Adult Nursing, Faculty of Nursing, Khon Kaen University, Khon Kaen, Thailand, 40002

E-Mail: oratsu@kku.ac.th

List of Supplementary information

Supplementary S1: PRISMA checklist.

Supplementary S2: Classification and comparison of bowel preparation educational, innovative technology interventions in the included trials.

Supplementary S3: Search strategy for each online database.

Supplementary S4: List of included studies.

Supplementary S5: List of excluded studies with their exclusion reasons.

Supplementary S6: List of ongoing studies.

Supplementary S7: Characteristics of included studies.

Supplementary S8: Study setting of included studies.

Supplementary S9: Bowel preparation information that contained in each intervention.

Supplementary S10: Outcomes reported in each included study.

Supplementary S11: Forest plots of subgroup analyses of the effects of bowel preparation innovative technology instructions (BPITIs) on adherence to overall instruction, purgative intake, and dietary restrictions when compared with routine care by types of bowel preparation intervention.

Supplementary S12: Forest plots of subgroup analyses of the effects of bowel preparation innovative technology instructions (BPITIs) on adequate bowel preparation and quality of bowel preparation score when compared with routine care by types of bowel preparation intervention.

Supplementary S13: Forest plots of subgroup analyses of the effects of bowel preparation innovative technology instructions (BPITIs) on quality of bowel preparation score when compared with routine care by types of bowel preparation scale.

Supplementary S14: Forest plots of the effects of bowel preparation innovative technology instructions (BPITIs) on adequate bowel preparation adjusted for confounding factors and secondary outcomes when compared with routine care.

Supplementary S15: Funnel plots of original and sensitivity analyses for adequate bowel preparation and quality of bowel cleansing score outcomes.

Supplementary S16: Summary of findings table for primary outcomes with reasons for grading the evidence.

Supplementary S1: PRISMA checklist.

Section and Topic	Item #	Checklist item	Location where item is reported
**TITLE**			
Title	1	Identify the report as a systematic review.	Page 1
**ABSTRACT**			
Abstract	2	See the PRISMA 2020 for Abstracts checklist.	Page 1
**INTRODUCTION**			
Rationale	3	Describe the rationale for the review in the context of existing knowledge.	Page 1-2
Objectives	4	Provide an explicit statement of the objective(s) or question(s) the review addresses.	Page 2
**METHODS**			
Eligibility criteria	5	Specify the inclusion and exclusion criteria for the review and how studies were grouped for the syntheses.	Page 2
Information sources	6	Specify all databases, registers, websites, organisations, reference lists and other sources searched or consulted to identify studies. Specify the date when each source was last searched or consulted.	Page 2-3
Search strategy	7	Present the full search strategies for all databases, registers and websites, including any filters and limits used.	Supple-mentary S3
Selection process	8	Specify the methods used to decide whether a study met the inclusion criteria of the review, including how many reviewers screened each record and each report retrieved, whether they worked independently, and if applicable, details of automation tools used in the process.	Page 3
Data collection process	9	Specify the methods used to collect data from reports, including how many reviewers collected data from each report, whether they worked independently, any processes for obtaining or confirming data from study investigators, and if applicable, details of automation tools used in the process.	Page 3
Data items	10a	List and define all outcomes for which data were sought. Specify whether all results that were compatible with each outcome domain in each study were sought (e.g. for all measures, time points, analyses), and if not, the methods used to decide which results to collect.	Page 2
	10b	List and define all other variables for which data were sought (e.g. participant and intervention characteristics, funding sources). Describe any assumptions made about any missing or unclear information.	Page 3
Study risk of bias assessment	11	Specify the methods used to assess risk of bias in the included studies, including details of the tool(s) used, how many reviewers assessed each study and whether they worked independently, and if applicable, details of automation tools used in the process.	Page 3-4
Effect measures	12	Specify for each outcome the effect measure(s) (e.g. risk ratio, mean difference) used in the synthesis or presentation of results.	Page 4
Synthesis methods	13a	Describe the processes used to decide which studies were eligible for each synthesis (e.g. tabulating the study intervention characteristics and comparing against the planned groups for each synthesis (item #5)).	Page 4
	13b	Describe any methods required to prepare the data for presentation or synthesis, such as handling of missing summary statistics, or data conversions.	Pages 4
	13c	Describe any methods used to tabulate or visually display results of individual studies and syntheses.	Pages 4
	13d	Describe any methods used to synthesize results and provide a rationale for the choice(s). If meta-analysis was performed, describe the model(s), method(s) to identify the presence and extent of statistical heterogeneity, and software package(s) used.	Pages 4

Section and Topic	Item #	Checklist item	Location where item is reported
	13e	Describe any methods used to explore possible causes of heterogeneity among study results (e.g. subgroup analysis, meta-regression).	Page 4
	13f	Describe any sensitivity analyses conducted to assess robustness of the synthesized results.	Page 4
Reporting bias assessment	14	Describe any methods used to assess risk of bias due to missing results in a synthesis (arising from reporting biases).	Page 4
Certainty assessment	15	Describe any methods used to assess certainty (or confidence) in the body of evidence for an outcome.	Page 4-5
**RESULTS**			
Study selection	16a	Describe the results of the search and selection process, from the number of records identified in the search to the number of studies included in the review, ideally using a flow diagram.	Page 5 and Figure 1
	16b	Cite studies that might appear to meet the inclusion criteria, but which were excluded, and explain why they were excluded.	Page 5 and Supple-mentary S5
Study characteristics	17	Cite each included study and present its characteristics.	Page 5-6 and Suppleme ntary S4, S7-S10
Risk of bias in studies	18	Present assessments of risk of bias for each included study.	Page 6 and Figure 3
Results of individual studies	19	For all outcomes, present, for each study: (a) summary statistics for each group (where appropriate) and (b) an effect estimate and its precision (e.g. confidence/credible interval), ideally using structured tables or plots.	Figure 4-6, and Supple-mentary S11-S14
Results of syntheses	20a	For each synthesis, briefly summarise the characteristics and risk of bias among contributing studies.	Page 6-10
	20b	Present results of all statistical syntheses conducted. If meta-analysis was done, present for each the summary estimate and its precision (e.g. confidence/credible interval) and measures of statistical heterogeneity. If comparing groups, describe the direction of the effect.	Page 6-10
	20c	Present results of all investigations of possible causes of heterogeneity among study results.	Page 6-9, Figure 6, Table 1, and Supple-mentary S11-S13
	20d	Present results of all sensitivity analyses conducted to assess the robustness of the synthesized results.	Page 6-9 and Table 2
Reporting biases	21	Present assessments of risk of bias due to missing results (arising from reporting biases) for each synthesis assessed.	Page 6-9 and Supple-mentary S15

Section and Topic	Item #	Checklist item	Location where item is reported
Certainty of evidence	22	Present assessments of certainty (or confidence) in the body of evidence for each outcome assessed.	Page 6-9, Figure 2, and Supple-mentary S16
**DISCUSSION**			
Discussion	23a	Provide a general interpretation of the results in the context of other evidence.	Page 10
	23b	Discuss any limitations of the evidence included in the review.	Page 13
	23c	Discuss any limitations of the review processes used.	Page 13-14
	23d	Discuss implications of the results for practice, policy, and future research.	Page 14
**OTHER INFORMATION**			
Registration and protocol	24a	Provide registration information for the review, including register name and registration number, or state that the review was not registered.	Abstract and Page 2
	24b	Indicate where the review protocol can be accessed, or state that a protocol was not prepared.	Page 2
	24c	Describe and explain any amendments to information provided at registration or in the protocol.	-
Support	25	Describe sources of financial or non-financial support for the review, and the role of the funders or sponsors in the review.	Page 16
Competing interests	26	Declare any competing interests of review authors.	Page 16
Availability of data, code and other materials	27	Report which of the following are publicly available and where they can be found: template data collection forms; data extracted from included studies; data used for all analyses; analytic code; any other materials used in the review.	Page 14

From: Page MJ, McKenzie JE, Bossuyt PM, Boutron I, Hoffmann TC, Mulrow CD, et al. The PRISMA 2020 statement: an updated guideline for reporting systematic reviews. BMJ 2021;372:n71. doi: 10.1136/bmj.n71

Supplementary S2: Classification and comparison of bowel preparation educational, innovative technology interventions in the included trials.

Interventions	Description
Computer-based education	Educating patients regarding bowel preparation and colonoscopy using a combination of multimedia on a computer-based platform.
Web-based education	Educating patients regarding bowel preparation and colonoscopy using a combination of multimedia on a web-based platform that they are able to access via a unique link.
Mobile apps or mobile application	Using a novel application (e.g., KakaoTalk, Whatsapp, Wechat, Moviprep, and Prepit Ferring BV.) to provide information related to bowel preparation and colonoscopy that patients are able to access via their mobile phones. In addition, there are several functions of reminder and communication between healthcare providers and patients within the apps, including notifications to remind patients, chat, and features to track patients' progression.
Short message service (SMS)	Sending repeated short text-message to patients' mobile phones in order to remind the scheduled colonoscopy appointment time, deliver bowel preparation and colonoscopy information, answer questions, and encourage patients to contact the professionals if they have any questions about bowel preparation.
Telephone call	Calling patients to emphasize the scheduled colonoscopy appointment time, the importance of bowel preparation, the purgative intake protocol and side effects, the adequate diet consumption, and encouraging patients to contact the professionals if they have any questions about bowel preparation.
VDO stream from on-site hospital devices	Providing patients with an educational video regarding colonoscopy and bowel preparation information. Individuals can access the educational video via hospital devices (e.g., iPads, tablets, laptops, or personal computers) located in a separate room at hospitals or outpatient clinics.
VDO stream from personal devices	Providing patients with an educational video regarding colonoscopy and bowel preparation contents. Individuals can view the educational video using their own devices (e.g., iPads, tablets, laptops, or personal computers) by clicking on a link that directly links to a website containing the video. Patients can view the video as many times as they prefer.

Supplementary S3: Search strategy for each online database.

1. PubMed

Search	Query
#1	colonoscope[MH]
#2	colonoscop* OR colonscop*
#3	#1 OR #2
#4	bowel preparation solutions[MH]
#5	intestine[MH]
#6	"intestine" OR "intestinal" OR "bowel"
#7	#5 OR #6
#8	("preparation" OR "preparations" OR "prep" OR "prepare" OR "prepares" OR "prepared" OR "preparing") OR ("clear" OR "clearing" OR "clean" OR "cleaning" OR "cleansing")
#9	#7 AND #8
#10	cathartic* OR "bowel evacuant*" OR purgative*
#11	#4 OR #9 OR #10
#12	educational technologies[MH]
#13	educat* OR instruct* OR teach* OR counsel* OR coach* OR mentor* OR initiat* OR lectur* OR tutor* OR learn*
#14	"multimedia" OR "digital" OR "eHealth" OR "mHealth" OR ("media" AND "delivered") OR "computer" OR "internet" OR "web-based" OR "social media" OR "blog" OR "Facebook"
#15	"mobile device*" OR smartphone* OR "smart phone*" OR tablet* OR computer* OR "cell phone*" OR "application" OR "apps" OR "app" OR "software" OR "iPad" OR "iPhone"
#16	"Social media" OR "Facebook" OR "twitter" OR "Instagram"
#17	"VDO" OR video* OR "optical storage device*" OR "VCD" OR "digital video disk" OR "DVD" OR movie* OR "YouTube" OR "animation" OR "anime"
#18	"tape record*" OR "audiovisual*" OR "audio-visual*" OR "audio sound" OR podcast*
#19	(book* AND illustrat*) OR ("medical illustrat*") OR "motion picture*" OR (model* AND "structural") OR "ebook" OR "e-book"
#20	"short message service" OR "SMS" OR "multimedia messaging service" OR "MMS" OR "chat bot*" OR "chat-bot*" OR chatbot* OR email* OR "e-mail*" OR "Wechat"
#21	#12 OR (#13 AND (#14 OR #15 OR #16 OR #17 OR #18 OR #19 OR #20))
#22	#11 AND #21
#23	randomized controlled trial[PT] OR controlled clinical trial[PT]
#24	(("randomized" OR "randomised") AND "controlled trial*") OR (("randomized" OR "randomised") AND "control trial*") OR "RCT" OR "controlled clinical trials" OR "randomly"
#25	#23 OR #24
#26	#3 AND #22 AND #25

2. CENTRAL

Search	Query
#1	MeSH descriptor: [Colonoscopes] explode all trees
#2	colonoscop* OR colonscop*
#3	#1 OR #2
#4	MeSH descriptor: [Cathartics] explode all trees
#5	MeSH descriptor: [Intestines] explode all trees
#6	"intestine" OR "intestinal" OR "bowel"
#7	#5 OR #6
#8	("preparation" OR "preparations" OR "prep" OR "prepare" OR "prepares" OR "prepared" OR "preparing") OR ("clear" OR "clearing" OR "clean" OR "cleaning" OR "cleansing")
#9	#7 AND #8
#10	cathartic* OR ("bowel" NEAR/1 evacuant*) OR purgative*
#11	#4 OR #9 OR #10
#12	MeSH descriptor: [Educational Technology] explode all trees
#13	educat* OR instruct* OR teach* OR counsel* OR coach* OR mentor* OR initiat* OR lectur* OR tutor* OR learn*
#14	"multimedia" OR "digital" OR "eHealth" OR "mHealth" OR ("media" NEAR/1 "delivered") OR "computer" OR "internet" OR ("web" NEAR/1 "based") OR "social media" OR "blog" OR "Facebook"
#15	("mobile" NEAR/1 device*) OR smartphone* OR ("smart" NEAR/1 phone*) OR tablet* OR computer* OR ("cell" NEAR/1 phone*) OR "application" OR "apps" OR "app" OR "software" OR "iPad" OR "iPhone"
#16	"Social media" OR "Facebook" OR "twitter" OR "Instagram"
#17	"VDO" OR video* OR ("optical" NEAR/1 "storage" NEAR/1 device*) OR "VCD" OR "digital video disk" OR "DVD" OR movie* OR "YouTube" OR "animation" OR "anime"
#18	("tape" NEAR/1 record*) OR audiovisual* OR ("audio" NEAR/1 visual*) OR "audio sound" OR podcast*
#19	(book* NEAR/1 illustrat*) OR ("medical" NEAR/1 illustrat*) OR ("motion" NEAR/1 picture*) OR (model* NEAR/1 "structural") OR "ebook"
#20	"short message service" OR "SMS" OR "multimedia messaging service" OR "MMS" OR ("chat" NEAR/1 bot*) OR chatbot* OR "Wechat"
#21	#12 OR (#13 AND (#14 OR #15 OR #16 OR #17 OR #18 OR #19 OR #20))
#22	#11 AND #21
#23	#3 AND #22
#24	#3 AND #22 in Trials

3. Ovid (Medline)

Search	Query
#1	exp Colonoscopes/ or Colonoscopes.mp.
#2	(colonoscop$ or colonscop$).af.
#3	#1 OR #2
#4	exp Cathartics/ or Cathartics.mp.
#5	exp Intestines/ or Intestines.mp.
#6	("intestine" or "intestinal" or "bowel").af.
#7	#5 OR #6
#8	("preparation" or "preparations" or "prep" or "prepare" or "prepares" or "prepared" or "preparing").af.
#9	("clear" or "clearing" or "clean" or "cleaning" or "cleansing").af.
#10	8 or 9
#11	7 and 10
#12	(cathartic$ or bowel evacuant$ or purgative$).af.
#13	4 or 11 or 12
#14	exp Educational Technology/ or Educational Technology.mp.
#15	(educat$ or instruct$ or teach$ or counsel$ or coach$ or mentor$ or initiat$ or lectur$ or tutor$ or learn$).af.
#16	("multimedia" or "digital" or "eHealth" or "mHealth" or ("media" and "delivered") or "computer" or "internet" or "web-based" or "social media" or "blog" or "Facebook").af.
#17	(mobile device$ or smartphone$ or smart phone$ or tablet$ or computer$ or cell phone$ or "application" or "apps" or "app" or "software" or "iPad" or "iPhone").af.
#18	("Social media" or "Facebook" or "twitter" or "Instagram").af.
#19	("VDO" or video$ or optical storage device$ or "VCD" or "digital video disk" or "DVD" or television$ or movie$ or "YouTube" or "animation" or "anime").af.
#20	(tape record$ or audiovisual$ or audio-visual$ or "audio sound" or podcast$).af.
#21	((book$ and illustrat$) or medical illustrat$ or motion picture$ or (model$ and "structural") or "ebook" or "e-book").af.
#22	("short message service" or "SMS" or "multimedia messaging service" or "MMS" or chat bot$ or chat-bot$ or chatbot$ or email$ or e-mail$ or "Wechat").af.
#23	16 or 17 or 18 or 19 or 20 or 21 or 22
#24	15 and 23
#25	14 or 24
#26	randomized controlled trial.pt.
#27	controlled clinical trial.pt.
#28	((("randomized" or "randomised") and controlled trial$) or (("randomized" or "randomised") and control trial$) or "RCT" or "controlled clinical trials" or "randomly").af.
#29	26 or 27 or 28
#30	3 and 25 and 29

4. SCOPUS

Search	Query
#1	TITLE-ABS-KEY(colonoscop* OR colonscop*)
#2	TITLE-ABS-KEY("intestine" OR "intestinal" OR "bowel")
#3	TITLE-ABS-KEY(("preparation" OR "preparations" OR "prep" OR "prepare" OR "prepares" OR "prepared" OR "preparing") OR ("clear" OR "clearing" OR "clean" OR "cleaning" OR "cleansing"))
#4	#2 AND #3
#5	TITLE-ABS-KEY(cathartic* OR ("bowel" W/1 evacuant*) OR purgative*)
#6	#4 OR #5
#7	TITLE-ABS-KEY(educat* OR instruct* OR teach* OR counsel* OR coach* OR mentor* OR initiat* OR lectur* OR tutor* OR learn*)
#8	TITLE-ABS-KEY("multimedia" OR "digital" OR "eHealth" OR "mHealth" OR ("media" AND "delivered") OR "computer" OR "internet" OR "web-based" OR "social media" OR "blog" OR "Facebook")
#9	TITLE-ABS-KEY(("mobile" W/1 device*) OR smartphone* OR ("smart" W/1 phone*) OR tablet* OR computer* OR ("cell" W/1 phone*) OR "application" OR "apps" OR "app" OR "software" OR "iPad" OR "iPhone")
#10	TITLE-ABS-KEY("Social media" OR "Facebook" OR "twitter" OR "Instagram")
#11	TITLE-ABS-KEY("VDO" OR video* OR ("optical" W/1 "storage" W/1 device*) OR "VCD" OR "digital video disk" OR "DVD" OR movie* OR "YouTube" OR "animation" OR "anime")
#12	TITLE-ABS-KEY(("tape" W/1 record*) OR audiovisual* OR audio-visual* OR "audio sound" OR podcast*)
#13	TITLE-ABS-KEY((book* W/1 illustrat*) OR ("medical" W/1 illustrat*) OR ("motion" W/1 picture*) OR (model* W/1 "structural") OR "ebook" OR "e-book")
#14	TITLE-ABS-KEY("short message service" OR "SMS" OR "multimedia messaging service" OR "MMS" OR ("chat" W/1 bot*) OR chat-bot* OR chatbot* OR email* OR e-mail* OR "Wechat")
#15	#7 AND (#8 OR #9 OR #10 OR #11 OR #12 OR #13 OR #14)
#16	#6 AND #15
#17	#1 AND #16
#18	TITLE-ABS-KEY((("randomized" OR "randomised") AND (controlled W/1 trial*)) OR (("randomized" OR "randomised") AND (control W/1 trial*)) OR "RCT" OR "controlled clinical trials" OR "randomly")
#19	#17 AND #18

5. Web of science

Search	Query
#1	TS=(colonoscop* OR colonscop*)
#2	TS=("intestine" OR "intestinal" OR "bowel")
#3	TS=(("preparation" OR "preparations" OR "prep" OR "prepare" OR "prepares" OR "prepared" OR "preparing") OR ("clear" OR "clearing" OR "clean" OR "cleaning" OR "cleansing"))
#4	#2 AND #3
#5	TS=(cathartic* OR ("bowel" NEAR/1 evacuant*) OR purgative*)
#6	#4 OR #5
#7	TS=(educat* OR instruct* OR teach* OR counsel* OR coach* OR mentor* OR initiat* OR lectur* OR tutor* OR learn*)
#8	TS=("multimedia" OR "digital" OR "eHealth" OR "mHealth" OR ("media" AND "delivered") OR "computer" OR "internet" OR "web-based" OR "social media" OR "blog" OR "Facebook")
#9	TS=(("mobile" NEAR/1 device*) OR smartphone* OR ("smart" NEAR/1 phone*) OR tablet* OR computer* OR (cell NEAR/1 phone*) OR "application" OR "apps" OR "app" OR "software" OR "iPad" OR "iPhone")
#10	TS=("Social media" OR "Facebook" OR "twitter" OR "Instagram")
#11	TS=("VDO" OR video* OR ("optical" NEAR/1 "storage" NEAR/1 device*) OR "VCD" OR "digital video disk" OR "DVD" OR movie* OR "YouTube" OR "animation" OR "anime")
#12	TS=(("tape" NEAR/1 record*) OR audiovisual* OR audio-visual* OR "audio sound" OR podcast*)
#13	TS=((book* NEAR/1 illustrat*) OR ("medical" NEAR/1 illustrat*) OR ("motion" NEAR/1 picture*) OR (model* NEAR/1 "structural") OR "ebook" OR "e-book")
#14	TS=("short message service" OR "SMS" OR "multimedia messaging service" OR "MMS" OR ("chat" NEAR/1 bot*) OR chat-bot* OR chatbot* OR email* OR e-mail* OR "Wechat")
#15	(#8 OR #9 OR #10 OR #11 OR #12 OR #13 OR #14)
#16	#7 AND #15
#17	#6 AND #16
#18	#1 AND #17
#19	TS=((("randomized" OR "randomised") AND (controlled NEAR/1 trial*)) OR (("randomized" OR "randomised") AND (control NEAR/1 trial*)) OR "RCT" OR "controlled clinical trials" OR "randomly")
#20	#18 AND #19

6. CINAHL

Search	Query
#1	TI ((colonoscop* OR colonscop*)) OR AB ((colonoscop* OR colonscop*))
#2	TI (("intestine" OR "intestinal" OR "bowel")) OR AB (("intestine" OR "intestinal" OR "bowel"))
#3	TI ((("preparation" OR "preparations" OR "prep" OR "prepare" OR "prepares" OR "prepared" OR "preparing") OR ("clear" OR "clearing" OR "clean" OR "cleaning" OR "cleansing"))) OR AB ((("preparation" OR "preparations" OR "prep" OR "prepare" OR "prepares" OR "prepared" OR "preparing") OR ("clear" OR "clearing" OR "clean" OR "cleaning" OR "cleansing")))
#4	#2 AND #3
#5	TI ((cathartic* OR (bowel evacuant*) OR purgative*)) OR AB ((cathartic* OR (bowel evacuant*) OR purgative*))
#6	#4 OR #5
#7	TI ((educat* OR instruct* OR teach* OR counsel* OR coach* OR mentor* OR initiat* OR lectur* OR tutor* OR learn*)) OR AB ((educat* OR instruct* OR teach* OR counsel* OR coach* OR mentor* OR initiat* OR lectur* OR tutor* OR learn*))
#8	TI (("multimedia" OR "digital" OR "eHealth" OR "mHealth" OR ("media" AND "delivered") OR "computer" OR "internet" OR "web-based" OR "social media" OR "blog" OR "Facebook")) OR AB (("multimedia" OR "digital" OR "eHealth" OR "mHealth" OR ("media AND delivered") OR "computer" OR "internet" OR "web-based" OR "social media" OR "blog" OR "Facebook"))
#9	TI (((mobile device*) OR smartphone* OR (smart phone*) OR tablet* OR computer* OR (cell phone*) OR "application" OR "apps" OR "app" OR "software" OR "iPad" OR "iPhone")) OR AB (((mobile device*) OR smartphone* OR (smart phone*) OR tablet* OR computer* OR (cell phone*) OR "application" OR "apps" OR "app" OR "software" OR "iPad" OR "iPhone"))
#10	TI (("Social media" OR "Facebook" OR "twitter" OR "Instagram")) OR AB (("Social media" OR "Facebook" OR "twitter" OR "Instagram"))
#11	TI (("VDO" OR video* OR (optical storage device*) OR "VCD" OR "digital video disk" OR "DVD" OR movie* OR "YouTube" OR "animation" OR "anime")) OR AB (("VDO" OR video* OR (optical storage device*) OR "VCD" OR "digital video disk" OR "DVD" OR movie* OR "YouTube" OR "animation" OR "anime"))
#12	TI (((tape record*) OR audiovisual* OR audio-visual* OR "audio sound" OR podcast*)) OR AB (((tape record*) OR audiovisual* OR audio-visual* OR "audio sound" OR podcast*))
#13	TI (((book* AND illustrat*) OR (medical illustrat*) OR (motion picture*) OR (model* AND "structural") OR "ebook" OR "e-book")) OR AB (((book* AND illustrat*) OR (medical illustrat*) OR (motion picture*) OR (model* AND "structural") OR "ebook" OR "e-book"))
#14	TI (("short message service" OR "SMS" OR "multimedia messaging service" OR "MMS" OR (chat bot*) OR chat-bot* OR chatbot* OR email* OR e-mail* OR "Wechat")) OR AB (("short message service" OR "SMS" OR "multimedia messaging service" OR "MMS" OR (chat bot*) OR chat-bot* OR chatbot* OR email* OR e-mail* OR "Wechat"))
#15	(#8 OR #9 OR #10 OR #11 OR #12 OR #13 OR #14)
#16	#7 AND #15
#17	#6 AND #16
#18	#1 AND #17
#19	TI (((("randomized" OR "randomised") AND (controlled trial*)) OR (("randomized" OR "randomised") AND (control trial*)) OR "RCT" OR "controlled clinical trials" OR "randomly")) OR AB (((("randomized" OR "randomised") AND (controlled trial*)) OR (("randomized" OR "randomised") AND (control trial*)) OR "RCT" OR "controlled clinical trials" OR "randomly"))
#20	#18 AND #19

7. LILACS

Title, abstract, subject (((colonoscop* OR colonscop*) AND ((("intestine" OR "intestinal" OR "bowel") AND ("preparation" OR "preparations" OR "prep" OR "prepare" OR "prepares" OR "prepared" OR "preparing") OR ("clear" OR "clearing" OR "clean" OR "cleaning" OR "cleansing")) OR cathartic* OR ("bowel evacuant*" OR purgative*))) AND ((educat* OR instruct* OR teach* OR counsel* OR coach* OR mentor* OR initiat* OR lectur* OR tutor* OR learn*) AND (("multimedia" OR "digital" OR "eHealth" OR "mHealth" OR ("media" AND "delivered") OR "computer" OR "internet" OR "web-based" OR "social media" OR "blog" OR "Facebook") OR ("mobile device*" OR smartphone* OR "smart phone*" OR tablet* OR computer* OR "cell phone*" OR "application" OR "apps" OR "app" OR "software" OR "iPad" OR "iPhone") OR ("Social media" OR "Facebook" OR "twitter" OR "Instagram") OR ("VDO" OR video* OR "optical storage device*" OR "VCD" OR "digital video disk" OR "DVD" OR movie* OR "YouTube" OR "animation" OR "anime") OR ("tape record*" OR "audiovisual*" OR "audio-visual*" OR "audio sound" OR podcast*) OR ((book* AND illustrat*) OR ("medical illustrat*") OR "motion picture*" OR (model* AND "structural") OR "ebook" OR "e-book") OR ("short message service" OR "SMS" OR "multimedia messaging service" OR "MMS" OR "chat bot*" OR "chat-bot*" OR chatbot* OR email* OR "e-mail*" OR "Wechat"))) AND (((("randomized" OR "randomised") AND (controlled trial*)) OR (("randomized" OR "randomised") AND (control trial*)) OR "RCT" OR "controlled clinical trials" OR "randomly")))

8. OpenGrey

(colonoscop$ OR colonscop$) AND (("intestine" OR "intestinal" OR "bowel") OR (("preparation" OR "preparations" OR "prep" OR "prepare" OR "prepares" OR "prepared" OR "preparing") OR ("clear" OR "clearing" OR "clean" OR "cleaning" OR "cleansing")) OR (cathartic$ OR (bowel evacuant$) OR purgative$)) AND ((educat$ OR instruct$ OR teach$ OR counsel$ OR coach$ OR mentor$ OR initiat$ OR lectur$ OR tutor$ OR learn$) OR ("multimedia" OR "digital" OR "eHealth" OR "mHealth" OR ("media" AND "delivered") OR "computer" OR "internet" OR "web-based" OR "social media" OR "blog" OR "Facebook") OR ((mobile device$) OR smartphone$ OR (smart phone$) OR tablet$ OR computer$ OR (cell phone$) OR "application" OR "apps" OR "app" OR "software" OR "iPad" OR "iPhone") OR ("Social media" OR "Facebook" OR "twitter" OR "Instagram") OR ("VDO" OR video$ OR (optical storage device$) OR "VCD" OR "digital video disk" OR "DVD" OR television$ OR movie$ OR "YouTube" OR "animation" OR "anime") OR ((tape record$) OR audiovisual$ OR audio-visual$ OR "audio sound" OR podcast$) OR ((book$ AND illustrat$) OR (medical illustrat$) OR (motion picture$) OR (model$ AND "structural") OR "ebook" OR "e-book") OR ("short message service" OR "SMS" OR "multimedia messaging service" OR "MMS" OR (chat bot$) OR chat-bot$ OR chatbot$ OR email$ OR e-mail$ OR "Wechat")) AND ((("randomized" OR "randomised") AND (controlled trial$)) OR (("randomized" OR "randomised") AND (control trial$)) OR "RCT" OR "controlled clinical trials" OR "randomly")

9. Clinicaltrials.gov

colonoscopy | bowel prep educational

10. Google scholar

colonoscop* AND ("bowel" AND prep*) AND (education* AND technolog*) AND ("randomized" OR "randomised")

Supplementary S4: List of included studies.

**1.**	**Alvarez-Gonzalez, 2020**
	* Alvarez-Gonzalez MA, Pantaleón Sánchez MÁ, Bernad Cabredo B, Garciá-Rodríguez A, Frago Larramona S, Nogales O, et al. Educational nurse-led telephone intervention shortly before colonoscopy as a salvage strategy after previous bowel preparation failure: A multicenter randomized trial. Endoscopy. 2020;52(11):1026–35.<br>NCT03055689. Bowel Preparation for Repeat Colonoscopy After Preparation Failure (RepeatPrep Study). https://clinicaltrials.gov/show/NCT03055689. 2017.
**2.**	**Archer, 2021**
	* Archer T, Dear K, Foley S, Cole A, Andreyev J, Fateen W, et al. Does an educational video improve bowel preparation in patients first colonoscopy? A UK multi-centre RCT. Gut. 2021;70(SUPPL 1):A45-.<br>Archer T, Dear K, Thoufeeq M, Foley S, Cole A, Andreyev J, et al. Can an educational video improve the adequacy of bowel preparation for patients undergoing their first colonoscopy the ebops study. United Eur Gastroenterol J. 2021;9(SUPPL 8):831-832.<br>ISRCTN20368092. Improving bowel preparation for colonoscopy with the use of an educational video. http://www.who.int/trialsearch/Trial2.aspx?TrialID=ISRCTN20368092. 2019;
**3.**	**Back, 2018**
	* Back SY, Kim HG, Ahn EM, Park S, Jeon SR, Im HH, et al. Impact of patient audiovisual re-education via a smartphone on the quality of bowel preparation before colonoscopy: a single-blinded randomized study. Gastrointest Endosc. 2018;87(3):789-799.e4.<br>Kim YK, Kim HG, Back SY, Jeon SR, Kim JO, Lee JS, et al. Impact of audiovisual (AV) education by smart phone on the quality of bowel preparation before colonoscopy: preliminary report of a single-blinded randomized study. Gastrointest Endosc. 2017;85(5):AB196--AB196-.
**4.**	**Bearelly, 2014**
	* Bearelly D, Kottewar S, Almashhrawi A, Davis W, Marshall J, Ibdah J. Instructional video improves the quality of bowel preparation for colonoscopy. Am J Gastroenterol. 2014;109:S599--S599-.<br>NCT02013505. A validated educational video intervention improves the quality of bowel preparation for colonoscopy. https://clinicaltrials.gov/show/NCT02013505. 2013.
**5.**	**Bowman, 2014**
	Bowman EA, Benson ME, Hourigan J, Selby L, Caldera F. Double blinded randomized control trial to evaluate if an educational video can improve bowel preparation. Gastrointest Endosc. 2014;79(5):AB180–1.
**6.**	**Bytzer, 2007**
	Bytzer P, Lindeberg B. Impact of an information video before colonoscopy on patient satisfaction and anxiety - a randomized trial. Endoscopy. 2007;39(8):710–4.
**7.**	**Chen, 2021**
	* Chen G, Zhao Y, Xie F, Shi W, Yang Y, Yang A, et al. Educating outpatients for bowel preparation before colonoscopy using conventional methods vs virtual reality videos plus conventional methods: A randomized clinical trial. JAMA Netw Open. 2021;<br>NCT03667911. Virtual reality videos in improving bowel preparation quality of colonoscopy. https://clinicaltrials.gov/show/NCT03667911. 2018;<br>Zhao Y, Xie F, Bai X, Yang A, Wu D. Educational virtual reality videos in improving bowel preparation quality and satisfaction of outpatients undergoing colonoscopy: Protocol of a randomised controlled trial. BMJ Open. 2019;9(8).<br>Zhao Y, Xie F, Bai X, Yang A, Wu D. Effect of educational virtual reality videos on improving bowel preparation quality and satisfaction of outpatients undergoing colonoscopy. Turkish J Gastroenterol. 2019;30:S253–4.
**8.**	**Garg, 2016**
	* Garg S, Girotra M, Chandra L, Verma V, Kaur S, Allawy A, et al. Improved bowel preparation with multimedia education in a predominantly African-American population: A randomized study. Diagn Ther Endosc. 2016;2016.

	Garg S, Girotra M, Kaur S, Allawy A, Dutta S. Prospective randomized controlled trial to study the effect of multimedia education before screening colonoscopy on quality of bowel preparation and adenoma detection rate. Gastrointest Endosc. 2014;79(5):AB458–9.
**9.**	**Guo, 2020**
	* Guo B, Zuo X, Li Z, Liu J, Xu N, Li X, et al. Improving the quality of bowel preparation through an app for inpatients undergoing colonoscopy: a randomized controlled trial. J Adv Nurs. 2020;76(4):1037–45.<br><br>ChiCTR1900022748. Improving the quality of bowel preparation through an app for inpatients undergoing colonoscopy: a randomized controlled trial. http://www.who.int/trialsearch/Trial2.aspx?TrialID=ChiCTR1900022748. 2019;
**10.**	**Jeon, 2019**
	* Jeon SC, Kim JH, Kim SJ, Kwon HJ, Choi YJ, Jung K, et al. Effect of sending educational video clips via smartphone mobile messenger on bowel preparation before colonoscopy. Clin Endosc. 2019;52(1):53–8.<br><br>Kim JH, Jeon SC, Kwon HJ, Choi YJ, Kim SJ, Jung K, et al. The effect of sending educational video clips via smartphone mobile messenger on bowel preparation before colonoscopy. J Gastroenterol Hepatol. 2018;33:245--245-.
**11.**	**Jung, 2022**
	* Jung DH, Gweon TG, Lee SJ, Son NH, Kim BW, Huh CW. Combination of enhanced instructions improve quality of bowel preparation: A prospective, colonoscopist-blinded, randomized, controlled study. Dis Colon Rectum. 2022;65(1):117–24.<br><br>KCT0003184. The effect of enhanced education for bowel preparation using a combination of educational video and telephone based re-education: prospective, colonoscopist-blinded, randomized controlled study. http://www.who.int/trialsearch/Trial2.aspx?TrialID=KCT0003184. 2018;
**12.**	**Kang, 2016**
	* Kang X, Zhao L, Leung F, Luo H, Wang L, Wu J, et al. Delivery of instructions via mobile social media app increases quality of bowel preparation. Clin Gastroenterol Hepatol. 2016;14(3):429-435.e3.<br><br>NCT02140827. Instant messaging program (Wechat) improve the quality of bowel preparation. https://clinicaltrials.gov/show/NCT02140827. 2014;
**13.**	**Keswani, 2020**
	Keswani RN, Gregory DL, Wood M, Dolan NC, Chmiel R, Manka M, et al. Colonoscopy education delivered via the patient portal does not improve adherence to scheduled first-time screening colonoscopy. Endosc Int OPEN. 2020;8(3):E401–6.
**14.**	**Kim, 2014**
	Kim ES, Cho KB, Park KS, Choi ES, Lee SM, Kim ES, et al. Comparison of impact of reinforced education between telephone and short message service on the quality of bowel preparation: a prospective, colonoscopist-blinded, randomized, controlled study. J Gastroenterol Hepatol. 2014;2(1):A42--A42-.
**15.**	**Lam, 2021**
	* Lam TYT, Hui AJ, Sia F, Wong MY, Lee CCP, Chung KW, et al. Short Message Service reminders reduce outpatient colonoscopy nonattendance rate: A randomized controlled study. J Gastroenterol Hepatol. 2021;36(4):1044–50.<br><br>Lam YT, Hui AJ, Sia F, Ching JYL, Sung JJY. Can a short message service (SMS) reminder prior improve the quality of colonoscopy? J Gastroenterol Hepatol. 2015;31:305–6.
**16.**	**Lam, 2022**
	* Lam TYT, Wu PI, Tang RSY, Tse YK, Lau JYW, Wu JCY, et al. Nurse-led reinforced education by mobile messenger improves the quality of bowel preparation of colonoscopy in a population-based colorectal cancer screening program: A randomized controlled trial. Int J Nurs Stud. 2022;133:104301.<br><br>Lam TYT, Ching JYL, Tang RSY, Wu JCY, Sung JJY. Can a Whatsapp reminder improve the quality of colorectal cancer screening? a randomized controlled study. J Gastroenterol Hepatol. 2018;33:211--211-.<br><br>Lam TYT, Sung JJY, Wu J, Luk KCA. WhatsApp reminder improves the quality of colorectal cancer screening. a single blinded randomized controlled study. United Eur Gastroenterol J. 2019;7(8):836–7.<br><br>NCT03209739. WhatsApp reminder on bowel preparation level. https://clinicaltrials.gov/show/NCT03209739. 2017;

**17.**	**Lee, 2015**
	* Lee YJ, Kim ES, Choi JH, Lee KI, Park KS, Cho KB, et al. Impact of reinforced education by telephone and short message service on the quality of bowel preparation: A randomized controlled study. Endoscopy. 2015;47(11):1018–27.<br><br>NCT01911052. The impact of telephone and short message system based education on bowel preparation for colonoscopy. https://clinicaltrials.gov/show/NCT01911052. 2013;
**18.**	**Lee, 2016**
	Lee H, Lee S, Lee Y. The impact of patient education with smartphone application on the quality of bowel preparation for screening colonoscopy. United Eur Gastroenterol J. 2016 Oct 16;4(5_suppl):A157–720.
**19.**	**Liu, 2014**
	* Liu X, Luo H, Zhang L, Leung FW, Liu Z, Wang X, et al. Telephone-based re-education on the day before colonoscopy improves the quality of bowel preparation and the polyp detection rate: a prospective, colonoscopist-blinded, randomised, controlled study. Gut. 2014;63(1):125–30.<br><br>NCT01584817. A repeated instruction by telephone on the day before colonoscopy to patients undergoing colonoscopy. https://clinicaltrials.gov/show/NCT01584817. 2012;
**20.**	**Liu, 2018**
	Liu C, Song X, Hao H. Educational video followed by retelling bowel preparation process to improve colonoscopy bowel preparation quality: a prospective nursing intervention study. Med Sci Monit. 2018;24:6029–37.
**21.**	**Lorenzo-Zúñiga, 2015**
	* Lorenzo-Zuniga V, Moreno De Vega V, Marin I, Barbera M, Boix J, Lorenzo-Zúñiga V, et al. Improving the quality of colonoscopy bowel preparation using a smart phone application: a randomized trial. Dig Endosc. 2015;27(5):590–5.<br><br>NCT02303743. Improving bowel cleansing with a smart phone application. https://clinicaltrials.gov/show/NCT02303743. 2014;
**22.**	**Mahmud, 2019**
	Mahmud N, Doshi SD, Coniglio MS, Clermont M, Bernard D, Reitz C, et al. An automated text message navigation program improves the show rate for outpatient colonoscopy. Health Educ Behav. 2019;46(6):942–6.
**23.**	**Mahmud, 2021**
	Mahmud N, Asch DA, Sung J, Reitz C, Coniglio MS, McDonald C, et al. Effect of text messaging on bowel preparation and appointment attendance for outpatient colonoscopy: A randomized clinical trial. JAMA Netw Open. 2021;4(1):e2034553–e2034553.
**24.**	**Mi-Hee, 2017**
	Mi-Hee C, Jun-Ah S. Effects of video assisted education using smartphone on bowel preparation for colonoscopy. J Korean Acad Fundam Nurs. 2017;24(1):60–71.
**25.**	**Pan, 2015**
	Pan Y, Kang X, Luo H, Guo X. Instruction delivered by mobile social media app increases quality of bowel preparation and adenoma detection rate: a prospective, colonoscopist-blinded, randomised, controlled study. Gastrointest Endosc. 2015;81(5):AB146--AB146-.
**26.**	**Park, 2015**
	* Park J, Kim TO, Lee NY, Kim H, Seo EH, Heo NY, et al. The effectiveness of short message service to assure the preparation-to-colonoscopy interval before bowel preparation for colonoscopy. Gastroenterol Res Pract. 2015.
**27.**	**Park, 2016**
	* Park J-SS, Kim MS, Kim HK, Kim SI, Shin CH, Lee HJ, et al. A randomized controlled trial of an educational video to improve quality of bowel preparation for colonoscopy. BMC Gastroenterol. 2016;16(1):64.<br><br>KCT0001836. A randomized controlled trial of an educational video to improve quality of bowel preparation for colonoscopy. https://trialsearch.who.int/Trial2.aspx?TrialID=KCT0001836. 2016;
**28.**	**Patel, 2018**
	Patel V, Patel H, Cavanagh Y, Debari VA, Baddoura W. The role of mobile phone text message reminders in adherence to bowel preparation for outpatient colonoscopies. Gastroenterology. 2018;154(6):S-141-S-142.

**29.**	**Pearson, 2005**
	Pearson S, Maddern GJ, Hewett P. Interacting effects of preoperative information and patient choice in adaptation to colonoscopy. Dis Colon Rectum. 2005;48(11):2047–54.
**30.**	**Pillai, 2018**
	* Pillai A, Menon R, Oustecky D, Ahmad A. Educational colonoscopy video enhances bowel preparation quality and comprehension in an inner city population. J Clin Gastroenterol. 2018;52(6):515–8.<br><br>NCT02906969. Colonoscopy educational video upon inner city population. https://clinicaltrials.gov/show/NCT02906969 [Internet]. 2016; Available from: https://www.cochranelibrary.com/central/doi/10.1002/central/CN-01580317/full<br><br>Pillai A, Menon R, Oustecky D, Baffy N, Bui R, Kaufer S, et al. Educational colonoscopy video enhances bowel prep quality and comprehension in an inner city population. Gastrointest Endosc. 2013;77(5 SUPPL. 1):AB510.
**31.**	**Prakash, 2013**
	Prakash SR, Verma S, McGowan J, Smith BE, Shroff A, Gibson GH, et al. Improving the quality of colonoscopy bowel preparation using an educational video. J Can Gastroenterol [Canadian J Gastroenterol. 2013;27(12):696–700.
**32.**	**Rice, 2016**
	* Rice SC, Higginbotham T, Dean MJ, Slaughter JC, Yachimski PS, Obstein KL. Video on diet before outpatient colonoscopy does not improve quality of bowel preparation: A prospective, randomized, controlled trial. Am J Gastroenterol. 2016;111(11):1564–71<br><br>NCT02690779. Bowel preparation quality for screening colonoscopy. https://clinicaltrials.gov/show/NCT02690779. 2016.<br><br>Rice SC, Higginbotham T, Dean MJ, Slaughter JC, Yachimski PS, Obstein KL. Comprehensive patient education on diet prior to outpatient colonoscopy does not improve quality of bowel preparation: a prospective, randomized, controlled trial. Gastrointest Endosc. 2016;83(5):AB220–1.
**33.**	**Seoane, 2020**
	* Seoane A, Font X, Pérez JC, Pérez R, Enriquez CF, Parrilla M, et al. Evaluation of an educational telephone intervention strategy to improve non-screening colonoscopy attendance: A randomized controlled trial. World J Gastroenterol. 2020;26(47):7568–83.<br><br>NCT03458377. Telephone educational intervention by the gastrointestinal endoscopy nurse. global impact on the quality of colonoscopy. https://clinicaltrials.gov/ct2/show/NCT03458377?term=bowel+prep+educational&cond=colonoscopy&draw=2&rank=16. 2018;
**34.**	**Sharara, 2017**
	* Sharara AI, Chalhoub JM, Beydoun M, Shayto RH, Chehab H, Harb AH, et al. A customized mobile application in colonoscopy preparation: A randomized controlled trial. Clin Transl Gastroenterol. 2017;8(1).<br><br>Sharara AI, Chalhoub JM, Beydoun M, Harb AH, Chehab H, El Reda ZD, et al. The effectiveness of a customized mobile application in colonoscopy preparation: a randomized controlled trial. United Eur Gastroenterol J. 2015;3(5):A188--A188-.
**35.**	**Shaw, 2001**
	Shaw MJ, Beebe TJ, Tomshine PA, Adlis SA, Cass OW. A randomized, controlled trial of interactive, multimedia software for patient colonoscopy education. J Clin Gastroenterol. 2001;32(2):142–7.
**36.**	**Shi, 2019**
	Shi H, Chen S-Y, Huang H, Huang R, Jiang Y, Huang J-Y, et al. Impact of patient education on bowel preparation quality before water-aided colonoscopy. World Chinese J Dig. 2019 May 28;27(10):632–6.
**37.**	**Solonowicz, 2022**
	* Solonowicz O, Stier M, Kim K, Kupfer S, Tapper E, Sengupta N. Digital navigation improves no-show rates and bowel preparation quality for patients undergoing colonoscopy: A randomized controlled quality improvement study. J Clin Gastroenterol. 2022 Feb;56(2):166–72.<br><br>Solonowicz O, Stier MW, Kim K, Kupfer SS, Sengupta N. Digital navigation improves no-show rates and bowel preparation quality for patients undergoing colonoscopy - A randomized controlled study. Gastroenterology. 2020;158(6):S-176

38. Suzuki, 2018 a21

* Suzuki MM, Bardi M, Takach O, Galorport C, Yonge J, Harris N, et al. Randomized Prospective Study: Impact of the Patient Education Website on the Quality of Outpatient Bowel Preparation for Colonoscopy: J Can Assoc Gastroenterol. 2018;1(suppl_1):38–9.

NCT02466724. Study on the impact of education website on the quality of outpatient bowel preparation for colonoscopy. https://clinicaltrials.gov/ct2/show/NCT02466724?term=bowel+prep+educational&cond=colonoscopy&draw=3&rank=12. 2015;

39. van der Zander, 2021

van der Zander QEW, Reumkens A, van de Valk B, Winkens B, Masclee AAM, de Ridder RJJ. Effects of a personalized smartphone app on bowel preparation quality: Randomized controlled trial. JMIR mHealth uHealth. 2021;9(8):e26703.

40. Veldhuijzen, 2020

* Veldhuijzen G, Klemt-Kropp M, Terhaar sive Droste JS, van Balkom B, van Esch AAJ, Drenth JPH. Computer-based patient education is non-inferior to nurse counselling prior to colonoscopy: a multicenter randomized controlled trial. Endoscopy. 2021 Mar 23;53(03):254–63.

NCT02656602. Computer-assisted instruction before colonoscopy is as effective as nurse counselling, aAcontrolled trial. https://clinicaltrials.gov/show/NCT02656602. 2016;

41. Walker, 2022

* Walker TB, Hengehold TA, Garza K, Rogers BD, Early D. An interactive video educational tool does not improve the quality of bowel preparation for colonoscopy: A randomized controlled study. Dig Dis Sci. 2022;67(6):2347–57.

NCT04491565. An interactive video educational tool improves the quality of bowel preparation for colonoscopy: A randomized controlled study. https://clinicaltrials.gov/show/NCT04491565. 2020;

42. Walter, 2019

Walter B, Klare P, Strehle K, Aschenbeck J, Ludwig L, Dikopoulos N, et al. Improving the quality and acceptance of colonoscopy preparation by reinforced patient education with short message service: results from a randomized, multicenter study (PERICLES-II). Gastrointest Endosc. 2019;89(3):506-513.e4.

43. Walter, 2021

* Walter B, Frank R, Ludwig L, Dikopoulos N, Mayr M, Neu B, et al. Smartphone application to reinforce education increases high-quality preparation for colorectal cancer screening colonoscopies in a randomized trial. Clin Gastroenterol Hepatol. 2021;19(2):331-338.e5.

NCT03290157. Improvement of colonoscopy preparation by using a SPA. https://clinicaltrials.gov/show/NCT03290157. 2017;

Walter B, Frank R, Hann A, Ludwig L, Dikopoulos N, Neu B, et al. Effect of digital patient reinforcement with an smartphone app on high quality colonoscopy preparation in CRC screening; results from the multicenter Coloprapp study. Gastrointest Endosc. 2020;91(6):AB31--AB31-.

Walter B, Frank R, Ludwig L, Dikopoulos N, Hann A, Mayr M, et al. Impact of digital patient reinforcement on high quality colonoscopy preparation in CRC screening: results from the multi-center Colopr APP-study. United Eur Gastroenterol J. 2019;7(8):174--174-.

Walter B, Frank R, Ludwig L, Dikopoulos N, Mayr M, Bruno N, et al. Implications of reinforced education in high quality colonoscopy preparation using a smartphone application: results from the coloprappstudy. Endoscopy. 2019;51(4):S55--S55-.

44. Wang, 2019

* Wang S-LL, Wang Q, Yao J, Zhao S-BB, Wang L-SS, Li Z-SS, et al. Effect of WeChat and short message service on bowel preparation: an endoscopist-blinded, randomized controlled trial. Eur J Gastroenterol Hepatol. 2019;31(2):170–7.

NCT02832869. Impact of reinforced education by Wechat and short message service on the quality of bowel preparation. https://clinicaltrials.gov/show/NCT02832869. 2016;

Wang S-L, Wang Q, Zhao S-B, Bai Y, Li Z-S. Impact of reinforced education by WeChat and short message service on the quality of bowel preparation: an endoscopist-blinded, randomized controlled trial. J Dig Dis. 2017;18:36–7.

**45.**	**Wen, 2020**
	Wen M-C, Kau K, Huang S-S, Huang W-H, Tsai L-Y, Tsai T-Y, et al. Smartphone education improves embarrassment, bowel preparation, and satisfaction with care in patients receiving colonoscopy: A randomized controlled trail. Med. 2020;99(46):e23102–e23102.
**46.**	**Xu, 2021**
	Xu F, Lu S, Dong L, He Y, Li H, Tang J. Educational video on the Wechat platform can effectively improve the quality of bowel preparation: A prospective, randomized, controlled study. Gastroenterol Nurs. 2021;44(1):47–51.
**47.**	**Zhang, 2018**
	Zhang Q-X, Li J, Li Y, Lei C-H, Shang B-X, Guan X-S, et al. Effect of education by messaging software on the quality of bowel preparation for colonoscopy. Chin Med J (Engl). 2018;131(14):1750–2.

* The primary reference for the study

Supplementary S5: List of excluded studies with their exclusion reasons.

1.	Ait Errami A, Krati K. A randomized controlled trial of an educational video to improve quality of bowel preparation for colonoscopy. United Eur Gastroenterol J. 2020;8(8 SUPPL):784-.	Untrustworthiness findings<br><br>Concerns have been raised regarding plagiarism issue for this report.<br>This report appears to have a high degree of similarity in terms of reporting patterns and findings to Park's study, which is one of our included studies.<br><br>**Reference:**<br>Park J-SS, Kim MS, Kim HK, Kim SI, Shin CH, Lee HJ, et al. A randomized controlled trial of an educational video to improve quality of bowel preparation for colonoscopy. BMC Gastroenterol. 2016;16(1):64.
2.	Arabul M, Kandemir A, Celik M, Alper E, Akpinar Z, Aslan F, et al. Impact of an information video before colonoscopy on patient satisfaction and anxiety. Turk J Gastroenterol. 2012;23(5):523–9.	Wrong intervention
3.	ChiCTR1900024666. Improving the quality of bowel preparation through an app for inpatients undergoing colonoscopy: a randomized controlled trial. http://www.who.int/trialsearch/Trial2.aspx?TrialID=ChiCTR1900024666. 2019;	Duplicate record
4.	Hill EN, Suzuki MM, Galorport C, Yonge J, Amar J, Bressler B, et al. A new standard: an open-label trial examining the effectiveness of individualized web based colonoscopy preparation instruction. Gastrointest Endosc. 2018;87(6):AB522--AB522-.	Wrong study design
5.	Hsu W-F, Liang C-C, Lin C-K, Lee T-H, Chung C-S. A modified bowel preparation protocol improves the quality of bowel cleansing for colonoscopy. Adv Dig Med. 2016;3(3):144–7.	Wrong study design
6.	* Kakkar A, Jacobson BC. Failure of an Internet-based health care intervention for colonoscopy preparation: a caveat for investigators. JAMA Intern Med. 2013;173(14):1374–6.<br><br>NCT01099553. Improving bowel preparation with an educational video. https://clinicaltrials.gov/ct2/show/NCT01099553?cond=colonoscopy&intr=bowel+prep+educational&draw=2&rank=8. 2010.	Wrong study design<br><br>Wrong study design
7.	Lachter J, Pahk E, Shackelford E, Asulin R, Lewis N. Movie instructions can improve preparation for colonoscopy. Am J Gastroenterol. 2016;111(9):1367.	Wrong publication type
8.	Lam TYT, Hui AJ, Sia F, Ching JYL, Sung JJY. Can a short message service (SMS) reminder prior improve the quality of out-patient colonoscopy? J Gastroenterol Hepatol. 2015;30:185–6.	Duplicate record
9.	NCT01648504. Johns Hopkins interactive eGuide to colonoscopy and Ipad office education to improve colonoscopy. 2012; Available from: https://clinicaltrials.gov/ct2/show/NCT01648504?term=bowel+prep+educational&cond=colonoscopy&draw=2&rank=19	Wrong study design
10.	Park J, Kim TO. How can we achieve good compliance for bowel preparation? Clin Endosc. 2019;52(1):3–4.	Wrong publication type

11. Sheng LP, Han CQ, Nie C, Xu T, Zhang K, Li XJ, et al. Watching videos of colonoscopies and receiving interpretations reduce pain and anxiety while increasing the satisfaction of patients. Dig Dis Sci. 2021;66(2):541–6.	Wrong intervention
12. Umezawa S, Higurashi T, Uchiyama S, Sakai E, Ohkubo H, Endo H, et al. Visual distraction alone for the improvement of colonoscopy-related pain and satisfaction. World J Gastroenterol. 2015;21(15):4707–14.	Wrong intervention
13. Veldhuijzen G, van Esch AA, Klemt-Kropp M, Terhaar sive Droste JS, Drenth JPH. E-Patient Counseling Trial (E-PACO): Computer based education versus nurse counseling for patients to prepare for colonoscopy. J Vis Exp. 2019 Aug 1;89(150):S126--S126-.	Duplicate record
14. Veldhuijzen, Govert, Klemt-Kropp, Michael, Noomen, Casper, Van Esch, Aura, Tjwa, Eric, & Drenth, Joost. (2017). Computer-assisted instruction before colonoscopy is as effective as nurse counselling, a clinical pilot trial. *Endoscopy International Open*, *05*(08), E792–E797. https://doi.org/10.1055/s-0043-110813	Wrong study design
15. Veldhuijzen G, van Esch AA, Klemt-Kropp M, Terhaar sive Droste JS, Drenth JPH. E-Patient Counseling Trial (E-PACO): Computer based education versus nurse counseling for patients to prepare for Colonoscopy. J Vis Exp. 2019 Aug 1;89(150):S126--S126-.	Wrong study design
16. Yu D, Patel S. The development of a novel smart phone application and the quality of bowel preparation for colonoscopy, a randomized controlled trial protocol. Gastrointest Endosc. 2018;87(6):AB521--AB521-.	Wrong study design
17. Yu D, Patel S. The use of a novel smart phone application and the quality of bowel preparation for colonoscopy, a randomized controlled trial. Surg Endosc other Interv Tech. 2018;32(1):S189--S189-.	Wrong study design

Supplementary S6: List of ongoing studies.

1.	ChiCTR-IOR-17013141. Influence of an educational booklet based on smart phone application on the quality of bowel preparation for patients undergoing colonoscopy. http://www.chictr.org.cn/showprojen.aspx?proj=18932. 2017;
2.	ChiCTR1800016419. Application of teach-back combined with Wechat in health education of patients undergoing colonoscopy. http://www.chictr.org.cn/showprojen.aspx?proj=27886 . 2018;
3.	ChiCTR2000032630. Effect of mobile video education based on quick record code scanning on the Quality of Bowel Preparation before Colonoscopy: a randomized control trial. http://www.chictr.org.cn/showproj.aspx?proj=53250. 2020;
4.	ChiCTR2000039030. Effect of WeChat messaging on improving the quality of bowel preparation for outpatient colonoscopy: a randomized controlled trial. https://trialsearch.who.int/Trial2.aspx?TrialID=ChiCTR2000039030. 2020;
5.	CTRI/2019/08/020732. A clinical trial to study the benefit of a mobile app to guide patients preparing for colonoscopy in comparison to usual instructional handouts. http://www.who.int/trialsearch/Trial2.aspx?TrialID=CTRI/2019/08/020732. 2019;
6.	IRCT20161116030926N3. The effect of education with educational film and visual aid cards on the bowel preparation and satisfaction in patients under colonoscopy. http://www.who.int/trialsearch/Trial2.aspx?TrialID=IRCT20161116030926N3. 2018;
7.	NCT03185299. Impact of reinforced education by video on the quality of bowel preparation. https://clinicaltrials.gov/show/NCT03185299. 2017;
8.	NCT03200366. Comparing interventions to increase colorectal cancer screening. https://clinicaltrials.gov/show/NCT03200366. 2017;
9.	NCT03595904. Internet-based motivational interviewing for colonoscopy. https://clinicaltrials.gov/ct2/show/NCT03595904. 2018;
10.	NCT04054388. LINE re-education before colonoscopy to confirm optimal bowel cleansing. https://clinicaltrials.gov/show/NCT04054388. 2019;
11.	NCT04895813. The effect of education and telephone guidance at colonoscopy. https://clinicaltrials.gov/ct2/show/NCT04895813?term=bowel+prep+educational&cond=colonoscopy&draw=2&rank=17. 2021;
12.	NCT04898426. Effect of enhanced interactive text messages and phone instruction on the quality of bowel preparation for colonoscopy. https://clinicaltrials.gov/show/NCT04898426. 2021;
13.	NCT05041283. Impact of artificial intelligence-based patient reinforcement on quality of colonoscopy. https://clinicaltrials.gov/show/NCT05041283. 2021;
14.	TCTR20190928002. Smart education application for bowel preparation improve quality colonoscopy. http://www.who.int/trialsearch/Trial2.aspx?TrialID=TCTR20190928002. 2019;
15.	TCTR20210201002. Effectiveness of educational video for bowel preparation before out-patient colonoscopy. https://www.cochranelibrary.com/central/doi/10.1002/central/CN-02240259/full. 2021;

Supplementary S7: Characteristics of included studies.

ID	Author Year Setting	Publication type	Study design	Analytical approach	Indication	Dietary restrictions	Bowel preparation regimen	NPO time	Receiving intervention until colonoscopy	Bowel preparation scale	Treatment	Age	BMI	Previous colonoscopy	DM	HT
1	Alvarez-Gonzalez 2020 Spain	Full text	RCT	ITT	Investigation	Low-fiber diet	Split	2 hours	1-2 days	BBPS	Telephone call	64.4 (15.7) [±]	26.7 (7.1) [±]	51.9%	18.9%	Not specified
											Routine care	63.7 (17.6) [±]	27.1 (5.3) [±]	56.5%	24.0%	Not specified
2	Archer 2021 UK	Abstract	RCT	Not specified	Investigation	Not specified	Not specified	Not specified	Not specified	BBPS	VDO stream from personal devices	Not specified	Not specified	Not specified	Not specified	Not specified
											Routine care	Not specified	Not specified	Not specified	Not specified	Not specified
3	Back 2018 ROK	Full text	RCT	PP	Investigation	Low-fiber diet, Soft diet	Split	Not specified	3 days	BBPS	VDO stream from personal devices	55.4 (12.8) [§]	23.5 (2.9) [§]	38.1%	Not specified	Not specified
											Routine care	57.6 (13.1) [§]	23.7 (3.49) [§]	31.9%	Not specified	Not specified
4	Bearelly 2014 US	Abstract	RCT	Not specified	Not specified	Not specified	Split	Not specified	Not specified	BBPS	VDO stream from personal devices	Not specified	Not specified	Not specified	Not specified	Not specified
											Routine care	Not specified	Not specified	Not specified	Not specified	Not specified
5	Bowman 2014 Not specified	Abstract	RCT	Not specified	Investigation	Not specified	Not specified	Not specified	Not specified	BBPS	VDO stream from personal devices	Not specified	Not specified	Not specified	Not specified	Not specified
											Routine care	Not specified	Not specified	Not specified	Not specified	Not specified
6	Bytzer 2007 Denmark	Full text	RCT	PP	Not specified	Not specified	Not specified	Not specified	3-20 days	Not specified	VDO stream from an on-site hospital device	52.8 (18.2) [§]	Not specified	12.5%	Not specified	Not specified
											Routine care	53.4 (16.0) [§]	Not specified	15.6%	Not specified	Not specified
7	Chen 2021 China	Full text	RCT	ITT	Investigation	Low-fiber diet	Split	3-4 hours	14 days	BBPS	VDO stream from an on-site hospital device	52.6 (11.4) [§]	23.9 (3.5) [§]	0.0%	9.2%	24.9%
											Routine care	50.5 (12.5) [§]	23.4 (3.8)	0.0%	5.8%	19.1%
8	Garg 2016 US	Full text	RCT	Not specified	Investigation	Liquid diet	Split	≥ 2 hours	Not specified	AC	VDO stream from an on-site hospital device	59.3 (18.1) [§]	Not specified	35.4%	Not specified	Not specified
											Routine care	57.3 (19.4) [§]	Not specified	34.8%	Not specified	Not specified

ID	Author Year Setting	Publication type	Study design	Analytical approach	Indication	Dietary restrictions	Bowel preparation regimen	NPO time	Receiving intervention until colonoscopy	Bowel preparation scale	Treatment	Age	BMI	Previous colonoscopy	DM	HT
9	Guo 2020 China	Full text	RCT	ITT	Not specified	Low-fiber diet	Split	Not specified	1 day	BBPS	Mobile apps	Not specified	Not specified	69.0%	4.1%	8.3%
											Routine care	Not specified	Not specified	66.2%	5.4%	6.8%
10	Jeon 2022 ROK	Full text	RCT	Not specified	Investigation	Low-fiber diet, Soft diet	Split	≥ 2 hours	Not specified	OBPS	VDO stream from personal devices	46.7 (9.9) §	23.5 (3.0) §	52.1%	Not specified	Not specified
											Routine care	49.9 (9.6) §	24.5 (3.1) §	58.9%	Not specified	Not specified
11	Jung 2022 ROK	Full text	RCT	ITT	Not specified	Low-fiber diet, Soft diet	Split	Not specified	4 days	BBPS	VDO stream from personal devices	52.6 (11.1) §	23.9 (2.8) §	Not specified	7.7%	Not specified
											Telephone re-education	56.0 (12.4) §	24.1 (3.2) §	Not specified	10.9%	Not specified
											Routine care	55.1 (10.2) §	24.8 (16.1) §	Not specified	8.5%	Not specified
12	Kang 2016 China	Full text	RCT	ITT	Investigation	Liquid diet, Low-fiber diet	Split	5 hours	Not specified	OBPS	Mobile apps	44.4 (13.2) §	22.3 (3.4) §	Not specified	2.1%	7.2%
											Routine care	45.5 (13.0) §	22.4 (3.8) §	Not specified	1.6%	8.9%
13	Keswani 2020 US	Full text	RCT	ITT	Investigation	Not specified	Not specified	Not specified	14 days	BBPS	VDO stream from personal devices	57.8 (7.3) §	Not specified	Not specified	Not specified	Not specified
											Routine care	57.2 (7.0) §	Not specified	Not specified	Not specified	Not specified
14	Kim 2014 ROK	Abstract	RCT	Not specified	Investigation	Not specified	Not specified	Not specified	1 day	BBPS	Telephone call	Not specified	Not specified	Not specified	Not specified	Not specified
											SMS	Not specified	Not specified	Not specified	Not specified	Not specified
											Routine care	Not specified	Not specified	Not specified	Not specified	Not specified
15	Lam 2021 HK	Full text	RCT	PP	Investigation	Low-fiber diet	Split	Not specified	7-10 days	AC	SMS	Not specified	Not specified	Not specified	12.8%	28.8%
											Routine care	Not specified	Not specified	Not specified	12.6%	30.8%
16	Lam 2022 HK	Full text	RCT	ITT	Investigation	Liquid diet, Low-fiber diet	Split	Not specified	4 days	AC and BBPS	Mobile apps	65.1 (4.2) §	24.3 (3.6) §	Not specified	13.7%	39.4%
											Routine care	65.5 (4.6) §	23.9 (2.9) §	Not specified	13.2%	37.4%

ID	Author Year Setting	Publica-tion type	Study design	Analy-tical approach	Indication	Dietary restric-tions	Bowel preparation regimen	NPO time	Receiving intervention until colonoscopy	Bowel preparation scale	Treatment	Age	BMI	Previous colono-scopy	DM	HT
17	Lee 2015 ROK	Full text	RCT	PP	Investigation	Low-fiber diet, Soft diet	Split	> 2 hours	2 days	BBPS	Telephone call	46.0 (12.2) §	24.1 (3.2) §	52.4%	4.0%	10.3%
											SMS	45.7 (12.4) §	24.0 (3.2) §	45.7%	7.9%	15.0%
											Routine care	47.1 (11.8) §	24.5 (3.8) §	41.6%	5.1%	18.2%
18	Lee 2016 ROK	Abstract	RCT	Not specified	Investigation	Not specified	Not specified	Not specified	Not specified	BBPS	Mobile apps	Not specified	Not specified	Not specified	Not specified	Not specified
											Routine care	Not specified	Not specified	Not specified	Not specified	Not specified
19	Liu 2014 China	Full text	RCT	ITT	Investigation	Liquid diet	Single	> 4 hours	1 day	OBPS	Telephone call	44.8 (12.5) §	22.4 (3.2) §	Not specified	Not specified	Not specified
											Routine care	45.7 (12.6) §	22.8 (3.4) §	Not specified	Not specified	Not specified
20	Liu 2018 China	Full text	RCT	Not specified	Investigation	Liquid diet	Split	> 2 hours	1 day	OBPS	VDO stream from an on-site hospital device	55.1 (6.3) §	24.3 (3.5) §	61.9%	8.4%	Not specified
											Routine care	54.4 (8.6) §	24.6 (2.8) §	60.0%	6.3%	Not specified
21	Lorenzo-Zúñiga 2015 Spain	Full text	RCT	Not specified	Investigation	Low-fiber diet	Single	3 hours	Not specified	HCS	Mobile apps	48.3 (13.5) §	Not specified	33.4%	Not specified	Not specified
											Routine care	52.5 (14.0) §	Not specified	42.1%	Not specified	Not specified
22	Mahmud 2019 US	Full text	RCT	Not specified	Not specified	Liquid diet	Split	6 hours	7 days	Not specified	SMS	56 (48, 63) ¶	Not specified	Not specified	Not specified	43.0%
											Routine care	60.5 (53, 67) ¶	Not specified	Not specified	Not specified	60.0%
23	Mahmud 2021 US	Full text	RCT	Not specified	Investigation	Liquid diet	Split	6 hours	7 days	AC	SMS	56 (50, 64) ¶	28.4 (24.3, 34.2) ¶	Not specified	16.4%	44.7%
											Routine care	56 (49, 64) ¶	28.6 (24.8, 34.2) ¶	Not specified	16.4%	42.2%
24	Mi-Hee 2017 ROK	Full text	RCT	Not specified	Not specified	Not specified	Not specified	Not specified	Not specified	OBPS	VDO stream from an on-site hospital device	46.7 (9.4) §	Not specified	43.3%	Not specified	Not specified
											VDO stream from personal devices	46.0 (11.7) §	Not specified	58.6%	Not specified	Not specified
											Routine care	47.2 (8.9) §	Not specified	27.6%	Not specified	Not specified

ID	Author Year Setting	Publica-tion type	Study design	Analy-tical approach	Indication	Dietary restric-tions	Bowel preparation regimen	NPO time	Receiving intervention until colonoscopy	Bowel preparation scale	Treatment	Age	BMI	Previous colono-scopy	DM	HT
25	Pan 2015 China	Abstract	RCT	ITT	Not specified	Not specified	Not specified	Not specified	Not specified	OBPS	Mobile apps	Not specified	Not specified	Not specified	Not specified	Not specified
											Routine care	Not specified	Not specified	Not specified	Not specified	Not specified
26	Park 2015 ROK	Full text	RCT	Not specified	Investigation	Liquid diet, Low-fiber diet, Soft diet	Split	2 hours	6 hours	OBPS	SMS	53.7 (10.4) [§]	23.6 (2.6) [§]	44.9%	4.4%	Not specified
											Routine care	55.8 (12.3) [§]	23.2 (2.8) [§]	55.6%	6.7%	Not specified
27	Park 2016 ROK	Full text	RCT	Not specified	Investigation	Liquid diet	Split	Not specified	1 day	OBPS	VDO stream from personal devices	49.2 (8.6) [§]	24.3 (3.0) [§]	61.6%	8.8%	Not specified
											Routine care	47.3 (9.2) [§]	24.7 (3.4) [§]	56.7%	5.2%	Not specified
28	Patel 2018 US	Abstract	RCT	Not specified	Not specified	Not specified	Not specified	Not specified	Not specified	BBPS	SMS	Not specified	Not specified	Not specified	Not specified	Not specified
											Routine care	Not specified	Not specified	Not specified	Not specified	Not specified
29	Pearson 2005 AUS	Full text	RCT	PP	Not specified	Not specified	Not specified	Not specified	Not specified	Not applicable	Routine care, VDO stream from an on-site hospital device (Choice condition)	59.8 (13.5) [§]	Not specified	Not specified	Not specified	Not specified
											Routine care (Choice condition)	59.7 (14.6) [§]	Not specified	Not specified	Not specified	Not specified
											Routine care, VDO stream from an on-site hospital device (No-choice condition)	57.2 (15.4) [§]	Not specified	Not specified	Not specified	Not specified
											Routine care (No-choice condition)	58.6 (15.8)	Not specified	Not specified	Not specified	Not specified
30	Pillai 2018 US	Full text	RCT	Not specified	Investigation	Not specified	Single, Split	Not specified	Not specified	OBPS	VDO stream from an on-site hospital device	Not specified	Not specified	Not specified	Not specified	Not specified
											Routine care	Not specified	Not specified	Not specified	Not specified	Not specified

ID	Author Year Setting	Publication type	Study design	Analytical approach	Indication	Dietary restrictions	Bowel preparation regimen	NPO time	Receiving intervention until colonoscopy	Bowel preparation scale	Treatment	Age	BMI	Previous colonoscopy	DM	HT
31	Prakash 2013 US	Full text	RCT	Not specified	Not specified	Not specified	Split	5 hours	Not specified	OBPS	VDO stream from personal devices	Not specified	Not specified	38.8%	Not specified	Not specified
											Routine care	Not specified	Not specified	56.1%	Not specified	Not specified
32	Rice 2016 US	Full text	RCT	Not specified	Investigation	Liquid diet	Split	2 hours	2-3 days	BBPS	VDO stream from personal devices	60.1 (8.8) [§]	32.8 (20.9) [§]	55.0%	15.0%	Not specified
											Routine care	61.0 (7.9) [§]	30.9 (6.1)	50.0%	11.0%	Not specified
33	Seoane 2020 Spain	Full text	RCT	ITT	Investigation	Low-fiber diet	Split	4 hours	7 days	BBPS	Telephone call	59.1 (16.2) [§]	26.7 (4.7) [§]	45.4%	17.1%	Not specified
											Routine care	59.9 (16.0) [§]	26.9 (4.6) [§]	49.1%	16.8%	Not specified
34	Sharara 2017 LEB	Full text	RCT	Not specified	Not specified	Liquid diet, Low-fiber diet	Split	4 hours	Not specified	AC	Mobile apps	52.0 (13.0) [§]	Not specified	Not specified	Not specified	Not specified
											Routine care	55.0 (12.0) [§]	Not specified	Not specified	Not specified	Not specified
35	Shaw 2001 US	Full text	RCT	Not specified	Investigation	Not specified	Not specified	Not specified	3-7 days	Not applicable	VDO stream from an on-site hospital device	53.0 (15.3) [§]	Not specified	Not specified	Not specified	Not specified
											Routine care	53.8 (12.2) [§]	Not specified	Not specified	Not specified	Not specified
36	Shi 2019 China	Full text	RCT	Not specified	Not specified	Low-fiber diet	Split	1 hour	2 days	BBPS	Mobile apps	Not specified	Not specified	Not specified	Not specified	Not specified
											Routine care	Not specified	Not specified	Not specified	Not specified	Not specified
37	Solonowicz 2022 US	Full text	RCT	Not specified	Investigation	Liquid diet, Low-fiber diet	Split	2 hours	14 days	BBPS	SMS	57 (50, 65) [¶]	Not specified	79.0%	12.0%	31.0%
											Routine care	57 (50, 66) [¶]	Not specified	75.0%	14.0%	32.0%
38	Suzuki 2018 Canada	Abstract	RCT	Not specified	Not specified	Not specified	Not specified	Not specified	Not specified	BBPS	Web-based education	Not specified	Not specified	Not specified	Not specified	Not specified
											Reaflet	Not specified	Not specified	Not specified	Not specified	Not specified

ID	Author Year Setting	Publication type	Study design	Analytical approach	Indication	Dietary restrictions	Bowel preparation regimen	NPO time	Receiving intervention until colonoscopy	Bowel preparation scale	Treatment	Age	BMI	Previous colonoscopy	DM	HT
39	van der Zander 2021 NL	Full text	RCT	ITT	Investigation	Liquid diet, Low-fiber diet	Split	Not specified	Not specified	BBPS	Mobile apps	56.9 (10.8) [§]	26.1 (4.6) [§]	39.0%	Not specified	Not specified
											Routine care	57.1 (12.4) [§]	52.7 (3.6) [§]	43.0%	Not specified	Not specified
40	Veldhuijzen 2020 NL	Full text	RCT	PP	Not specified	Not specified	Split	Not specified	Not specified	BBPS	Computer-based education	56.0 (14.5) [§]	Not specified	51.5%	Not specified	Not specified
											Routine care	56.0 (14.5) [§]	Not specified	46.8%	Not specified	Not specified
41	Walker 2022 US	Full text	RCT	ITT	Not specified	Not specified	Split	Not specified	Not specified	BBPS	VDO stream from personal devices	57.6 (12.6) [§]	29.1 (6.3) [§]	57.7%	14.4%	Not specified
											Routine care	57.5 (11.1) [§]	29.1 (6.1) [§]	61.8%	14.7%	Not specified
42	Walter 2019 DEU	Full text	RCT	ITT	Investigation	Liquid diet, Low-fiber diet	Split	1 hour	4 days	BBPS	SMS	47.5 (13.6) [§]	25.6 [£]	35.0%	4.0%	Not specified
											Routine care	47.2 (14.8) [§]	25.4 [£]	41.0%	5.0%	Not specified
43	Walter 2021 DEU	Full text	RCT	PP	Investigation	Liquid diet, Low-fiber diet	Split	≥ 1 hours	4 days	BBPS	Mobile apps	55.0 (7.6) [§]	26.4 (4.5) [§]	42.7%	7.3%	23.2%
											Routine care	56.8 (9.3) [§]	26.1 (4.9) [§]	47.3%	9.9%	37.4%
44	Wang 2019 China	Full text	RCT	PP	Investigation	Liquid diet, Low-fiber diet	Split	≥ 4 hours	2 days	BBPS	Mobile apps	48.9 (13.0) [§]	23.8 (4.8) [§]	32.8%	1.6%	17.2%
											SMS	52.6 (12.7) [§]	23.8 (3.5) [§]	37.2%	3.9%	26.4%
											Routine care	51.5 (12.1) [§]	23.0 (3.2) [§]	40.2%	7.1%	19.7%
45	Wen 2020 Taiwan	Full text	RCT	Not specified	Not specified	Not specified	Not specified	Not specified	Not specified	AC	Mobile apps	46.3 (11.7) [§]	24.0 (5.0) [§]	Not specified	Not specified	Not specified
											Routine care	48.4 (14.1) [§]	25.4 (4.9) [§]	Not specified	Not specified	Not specified
46	Xu 2021 China	Full text	RCT	Not specified	Not specified	Low-fiber diet	Split	5-6 hours	1 day	BBPS	VDO stream from personal devices	51.9 (10.5) [§]	Not specified	Not specified	Not specified	Not specified
											Routine care	54.2 (10.3) [§]	Not specified	Not specified	Not specified	Not specified

ID	Author Year Setting	Publication type	Study design	Analytical approach	Indication	Dietary restrictions	Bowel preparation regimen	NPO time	Receiving intervention until colonoscopy	Bowel preparation scale	Treatment	Age	BMI	Previous colonoscopy	DM	HT
47	Zhang 2018 China	Full text	RCT	ITT	Investigation	Not specified	Split	Not specified	2 days	BBPS	Mobile apps	51.2 (18.5) §	21.9 (3.8) §	Not specified	5.1%	11.9%
											Routine care	50.7 (17.9) §	21.7 (4.1) §	Not specified	4.5%	15.2%

± median (IQR), § mean (SD), ¶ median (p25, p75), £ mean

DM: diabetes mellitus, HT: hypertension, RCT: Randomised controlled trial, ITT: intention-to-treat, PP: per protocol, BBPS: Boston Bowel Preparation Scale, AC: Aronchick scale, OBPS: Ottawa Bowel Preparation Scale, HCS: Harefield Cleansing Scale, AUS: Australia, DEU: Germany, HK: Hong Kong, LEB: Lebanon, NL: Netherlands, ROK: Republic of Korea, UK: United Kingdom, US: United States

Supplementary S8: Study setting of included studies*.

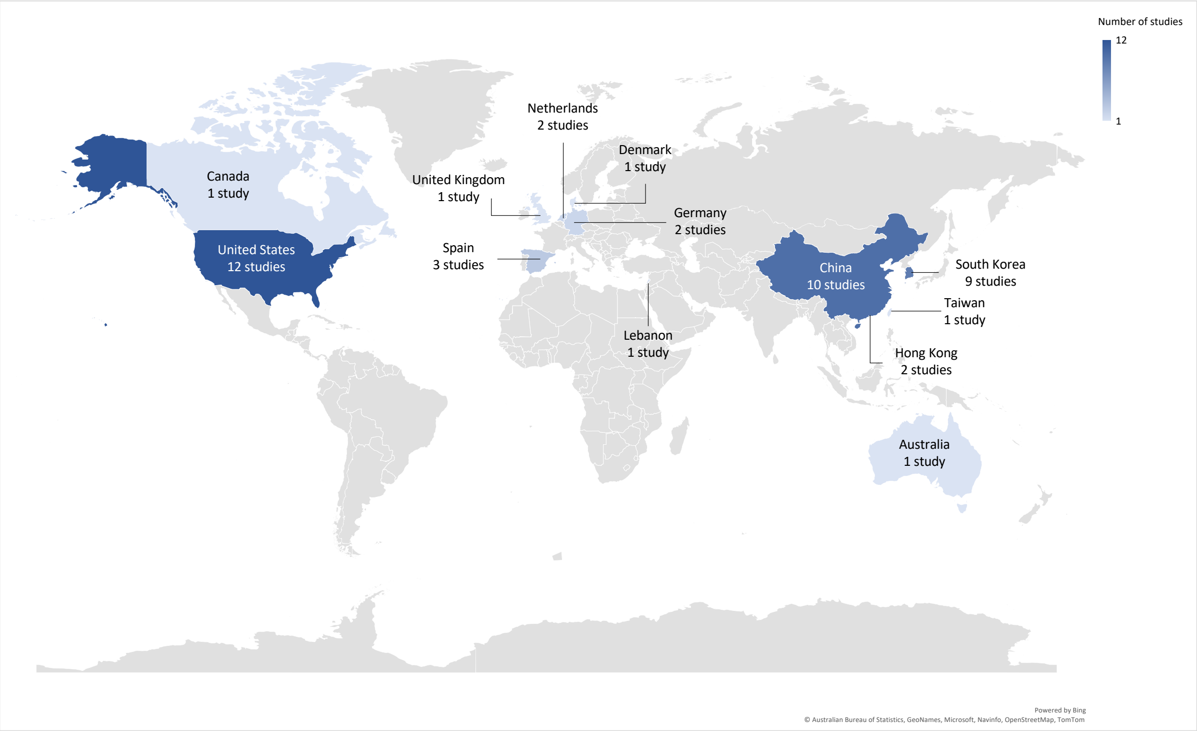

* This figure was generated by using Microsoft Excel version 16.73 (Build 23051401)

Supplementary S9: Bowel preparation information that contained in each intervention.

ID	Author	Intervention 1	Significance of BP	BP	Diet	Purgative	NPO	COL	Side effects	Intervention 2	Significance of BP	BP	Diet	Purgative	NPO	COL	Side effects	Intervention 3	Significance of BP	BP	Diet	Purgative	NPO	COL	Side effects
1	Alvarez-Gonzalez, 2020	Telephone call	/		/	/	/			Routine care	/		/	/	/										
2	Archer, 2021	VDO stream from personal devices								Routine care															
3	Back, 2018	VDO stream from personal devices	/	/	/	/	/		/	Routine care	/	/	/	/	/		/								
4	Bearelly, 2014	VDO stream from personal devices						/		Routine care						/									
5	Bowman, 2014	VDO stream from personal devices	/							Routine care															
6	Bytzer, 2007	VDO stream from an on-site hospital device	/	/	/	/	/	/	/	Routine care	/	/	/	/	/										
7	Chen, 2021	VDO stream from an on-site hospital device	/	/	/	/	/	/	/	Routine care	/	/	/	/	/										
8	Garg, 2016	VDO stream from an on-site hospital device	/	/	/	/	/	/	/	Routine care			/	/	/										
9	Guo, 2020	Mobile apps	/	/	/	/	/			Routine care				/	/										
10	Jeon, 2019	VDO stream from personal devices			/	/	/	/		Routine care			/	/	/	/									
11	Jung, 2022	VDO stream from personal devices	/	/	/	/				Telephone re-education			/	/				Routine care			/	/			
12	Kang, 2016	Mobile apps			/	/	/			Routine care			/	/	/										
13	Keswani, 2020	VDO stream from personal devices	/							Routine care	/														
14	Kim, 2014	Telephone call								SMS								Routine care							
15	Lam, 2021	SMS			/	/		/	/	Routine care			/	/		/	/								

ID	Author	Intervention 1	Significance of BP	BP	Diet	Purgative	NPO	COL	Side effects	Intervention 2	Significance of BP	BP	Diet	Purgative	NPO	COL	Side effects	Intervention 3	Significance of BP	BP	Diet	Purgative	NPO	COL	Side effects
16	Lam, 2022	Mobile apps	/		/	/			/	Routine care	/		/	/			/								
17	Lee, 2016	Mobile apps								Routine care															
18	Lee, 2015	Telephone call	/		/	/	/			SMS	/		/	/	/			Routine care	/		/	/	/		
19	Liu, 2014	Telephone call	/		/	/	/			Routine care	/		/	/	/										
20	Liu, 2018	VDO stream from an on-site hospital device	/		/	/	/			Routine care	/		/	/	/										
21	Lorenzo-Zúñiga, 2015	Mobile apps	/	/	/	/	/	/		Routine care	/	/	/	/	/	/									
22	Mahmud, 2019	SMS			/	/	/			Routine care			/	/	/										
23	Mahmud, 2021	SMS			/	/				Routine care			/	/											
24	Mi-Hee, 2017	VDO stream from an on-site hospital device	/	/	/	/	/	/		VDO stream from personal devices	/	/	/	/	/	/		Routine care	/	/	/	/	/	/	
25	Pan, 2015	Mobile apps								Routine care															
26	Park, 2015	SMS			/	/	/			Routine care			/	/	/										
27	Park, 2016	VDO stream from personal devices	/	/	/	/	/			Routine care	/	/	/	/	/										
28	Patel, 2018	SMS								Routine care															
29	Pearson, 2005	Routine care, VDO stream from an on-site hospital device (Choice condition)	/	/	/	/	/	/	/	Routine care (Choice condition)	/	/	/	/	/	/	/								
		Routine care, VDO stream from an on-site hospital device (No-choice condition)	/	/	/	/	/	/	/	Routine care (No-choice condition)	/	/	/	/	/	/	/								
30	Pillai, 2018	VDO stream from an on-site hospital device	/	/	/	/	/	/	/	Routine care	/	/	/	/	/	/	/								

ID	Author	Intervention 1	Significance of BP	BP	Diet	Purgative	NPO	COL	Side effects	Intervention 2	Significance of BP	BP	Diet	Purgative	NPO	COL	Side effects	Intervention 3	Significance of BP	BP	Diet	Purgative	NPO	COL	Side effects
31	Prakash, 2013	VDO stream from personal devices	/		/	/	/			Routine care	/		/	/	/										
32	Rice, 2016	VDO stream from personal devices	/		/	/	/			Routine care	/		/	/	/										
33	Seoane, 2020	Telephone call	/		/	/				Routine care			/	/											
34	Sharara, 2017	Mobile apps			/	/				Routine care			/	/											
35	Shaw, 2001	VDO stream from an on-site hospital device	/	/				/	/	Routine care															
36	Shi, 2019	Mobile apps	/		/	/	/			Routine care			/	/	/										
37	Solonowicz, 2022	SMS			/	/				Routine care															
38	Suzuki, 2018	Web-based education								Routine care															
39	van der Zander, 2021	Mobile apps	/		/	/		/		Routine care	/		/	/		/									
40	Veldhuijzen, 2020	Computer-based education	/	/	/	/	/	/	/	Routine care			/	/											
41	Walker, 2022	VDO stream from personal devices	/		/	/		/	/	Routine care	/		/	/		/	/								
42	Walter, 2019	SMS			/	/	/			Routine care			/	/	/										
43	Walter, 2021	Mobile apps	/	/	/	/	/			Routine care	/	/	/	/	/										
44	Wang, 2019	Mobile apps	/		/	/				SMS	/		/	/				Routine care	/		/	/			
45	Wen, 2020	Mobile apps	/	/		/		/	/	Routine care	/	/		/		/	/								
46	Xu, 2021	VDO stream from personal devices	/	/	/	/		/	/	Routine care	/	/	/	/		/	/								
47	Zhang, 2018	Mobile apps			/	/				Routine care			/	/											

Significance of BP: Clinical significance of bowel preparation, BP: Definition of bowel preparation, Purgative: Medications for bowel preparation, COL: Process of colonoscopic examination, Side effects: Side effects of colonoscopic examination

Supplementary S10: Outcomes reported in each included study.

ID	Author	Binary outcomes						Continuous outcomes					Total
		Adherence			**Bowel cleansing**	**Polyp detection**	**Satisfaction**	Adherence			**Bowel cleansing**	**Satisfaction**	
		Overall instruction	Purgative intake	Dietary restrictions				Overall instruction	Purgative intake	Dietary restrictions			
1	Alvarez-Gonzalez, 2020		/	/	/	/							**4**
2	Archer, 2021				/	/							**2**
3	Back, 2018								/	/	/	/	**4**
4	Bearelly, 2014				/	/					/		**3**
5	Bowman, 2014										/		**1**
6	Bytzer, 2007										/		**1**
7	Chen, 2021	/			/	/					/	/	**5**
8	Garg, 2016				/	/							**2**
9	Guo, 2020	/	/	/	/	/							**5**
10	Jeon, 2019	/			/	/					/		**4**
11	Jung, 2022	/			/	/					/	/	**5**
12	Kang, 2016	/	/	/	/	/					/		**6**
13	Keswani, 2020				/						/		**2**
14	Kim, 2014				/	/					/		**3**
15	Lam, 2021				/								**1**
16	Lam, 2022				/						/		**2**
17	Lee, 2016					/					/		**2**
18	Lee, 2015		/	/	/	/	/				/		**6**
19	Liu, 2014	/	/	/	/	/					/		**6**
20	Liu, 2018				/	/					/		**3**
21	Lorenzo-Zúñiga, 2015				/						/	/	**3**
22	Mahmud, 2019				/		/						**2**
23	Mahmud, 2021				/								**1**
24	Mi-Hee, 2017								/	/	/	/	**4**
25	Pan, 2015				/	/					/		**3**
26	Park, 2015		/	/	/	/					/		**5**
27	Park, 2016				/	/					/		**3**
28	Patel, 2018										/		**1**
29	Pearson, 2005											/	**1**
30	Pillai, 2018				/						/		**2**
31	Prakash, 2013						/				/		**2**
32	Rice, 2016				/						/		**2**
33	Seoane, 2020				/		/					/	**3**
34	Sharara, 2017	/	/	/	/						/	/	**6**
35	Shaw, 2001						/					/	**2**
36	Shi, 2019				/								**1**
37	Solonowicz, 2022				/						/	/	**3**
38	Suzuki, 2018				/								**1**
39	van der Zander, 2021				/	/					/	/	**4**
40	Veldhuijzen, 2020				/						/		**2**
41	Walker, 2022				/	/					/		**3**
42	Walter, 2019				/						/		**2**
43	Walter, 2021		/	/	/		/				/		**5**
44	Wang, 2019		/	/	/	/	/				/		**6**
45	Wen, 2020				/							/	**2**
46	Xu, 2021							/			/		**2**
47	Zhang, 2018	/		/	/						/		**4**
**Total**		**8**	**9**	**10**	**37**	**20**	**7**	**1**	**2**	**2**	**34**	**12**	**142**

Supplementary S11: Forest plots of subgroup analyses of the effects of bowel preparation innovative technology instructions (BPITIs) on adherence to overall instruction, purgative intake, and dietary restrictions when compared with routine care by types of bowel preparation intervention.

(A) adherence to overall instruction (binary outcome)

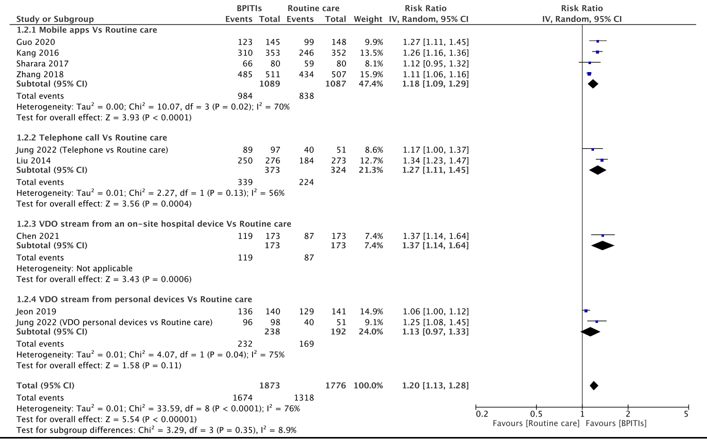

(B) adherence to overall instruction (continuous outcome)

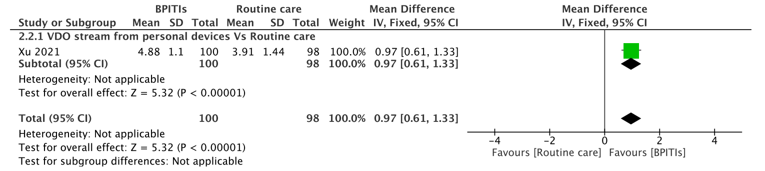

(C) adherence to purgative intake (binary outcome)

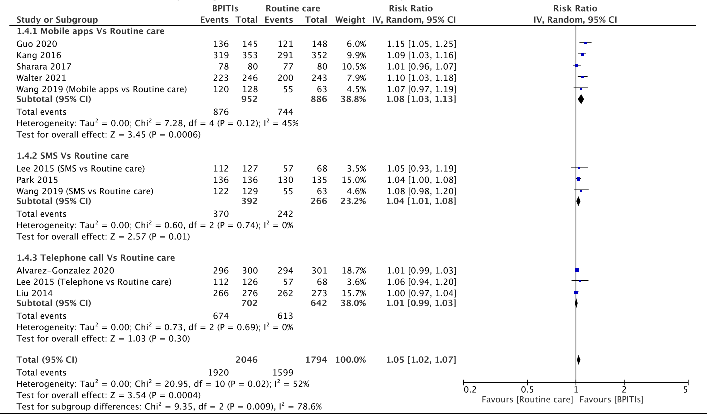

(D) adherence to purgative intake (continuous outcome)

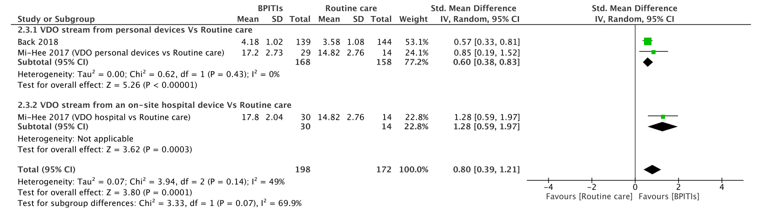

(E) adherence to dietary restrictions (binary outcome)

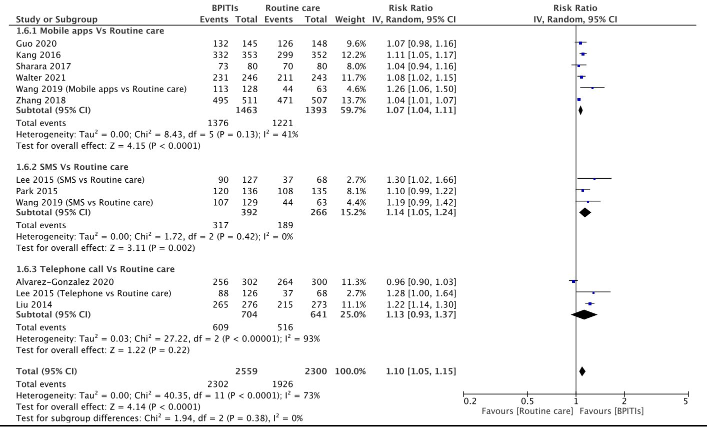

(F) adherence to dietary restrictions (continuous outcome)

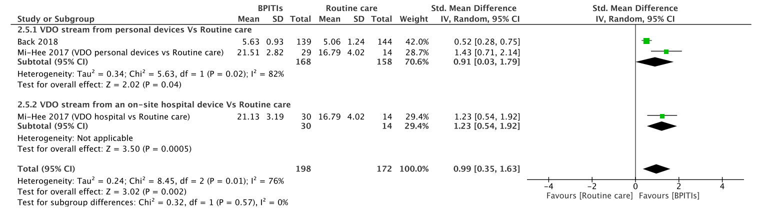

Supplementary S12: Forest plots of subgroup analyses of the effects of bowel preparation innovative technology instructions (BPITIs) on adequate bowel preparation and quality of bowel preparation score when compared with routine care by types of bowel preparation intervention.

(A) adequate bowel preparation

Study or Subgroup	BPITIs Events	BPITIs Total	Routine care Events	Routine care Total	Weight	Risk Ratio IV, Random, 95% CI
**1.8.1 Computer-based education Vs Routine care**						
Alvarez-Gonzalez 2020	249	303	237	302	3.0%	1.05 [0.97, 1.13]
Veldhuijzen 2020	261	280	204	217	3.7%	0.99 [0.95, 1.04]
**Subtotal (95% CI)**		**583**		**519**	**6.7%**	**1.01 [0.96, 1.06]**
Total events	510		441			
Heterogeneity: $Tau^2 = 0.00; Chi^2 = 1.37, df = 1 (P = 0.24); I^2 = 27\%$						
Test for overall effect: Z = 0.35 (P = 0.72)						
**1.8.2 Mobile apps Vs Routine care**						
Guo 2020	112	145	84	148	1.5%	1.36 [1.15, 1.61]
Kang 2016	318	387	266	383	2.9%	1.18 [1.09, 1.28]
Lam 2022	286	343	258	342	3.0%	1.11 [1.02, 1.19]
Lorenzo-Zúñiga 2015	108	108	146	152	3.9%	1.04 [1.00, 1.08]
Pan 2015	318	387	266	383	2.9%	1.18 [1.09, 1.28]
Sharara 2017	62	80	66	80	1.6%	0.94 [0.80, 1.10]
Shi 2019	188	200	174	200	3.3%	1.08 [1.01, 1.15]
van der Zander 2021	80	81	77	81	3.5%	1.04 [0.98, 1.10]
Walter 2021	227	246	202	243	3.2%	1.11 [1.04, 1.19]
Wang 2019 (Mobile apps vs Routine care)	114	127	41	62	1.3%	1.36 [1.13, 1.64]
Wen 2020	51	70	36	70	0.7%	1.42 [1.08, 1.85]
Zhang 2018	489	542	469	534	3.8%	1.03 [0.98, 1.07]
**Subtotal (95% CI)**		**2716**		**2678**	**31.8%**	**1.11 [1.06, 1.16]**
Total events	2353		2085			
Heterogeneity: $Tau^2 = 0.00; Chi^2 = 42.04, df = 11 (P < 0.0001); I^2 = 74\%$						
Test for overall effect: Z = 4.63 (P < 0.00001)						
**1.8.3 SMS Vs Routine care**						
Kim 2014 (SMS vs Routine care)	118	127	59	68	2.5%	1.07 [0.96, 1.19]
Lam 2021	687	983	665	1010	3.4%	1.06 [1.00, 1.13]
Lee 2015 (SMS vs Routine care)	119	126	59	67	2.6%	1.07 [0.97, 1.18]
Mahmud 2019	16	19	30	31	1.1%	0.87 [0.71, 1.07]
Mahmud 2021	226	242	238	262	3.6%	1.03 [0.98, 1.08]
Park 2015	108	136	78	135	1.5%	1.37 [1.16, 1.63]
Solonowicz 2022	540	608	470	538	3.7%	1.02 [0.97, 1.06]
Walter 2019	225	248	199	247	3.1%	1.13 [1.05, 1.21]
Wang 2019 (SMS vs Routine care)	111	128	41	62	1.3%	1.31 [1.08, 1.59]
**Subtotal (95% CI)**		**2617**		**2420**	**22.8%**	**1.08 [1.03, 1.13]**
Total events	2150		1839			
Heterogeneity: $Tau^2 = 0.00; Chi^2 = 25.09, df = 8 (P = 0.001); I^2 = 68\%$						
Test for overall effect: Z = 2.93 (P = 0.003)						
**1.8.4 Telephone call Vs Routine care**						
Jung 2022 (Telephone vs Routine care)	83	101	40	53	1.4%	1.09 [0.91, 1.30]
Kim 2014 (Telephone vs Routine care)	125	126	59	68	2.7%	1.14 [1.04, 1.26]
Lee 2015 (Telephone vs Routine care)	124	125	59	67	2.8%	1.13 [1.03, 1.23]
Liu 2014	249	305	211	300	2.7%	1.16 [1.06, 1.27]
Seoane 2020	622	673	567	627	3.9%	1.02 [0.99, 1.06]
**Subtotal (95% CI)**		**1330**		**1115**	**13.5%**	**1.10 [1.03, 1.18]**
Total events	1203		936			
Heterogeneity: $Tau^2 = 0.00; Chi^2 = 12.71, df = 4 (P = 0.01); I^2 = 69\%$						
Test for overall effect: Z = 2.81 (P = 0.005)						
**1.8.5 VDO stream from an on-site hospital device Vs Routine care**						
Chen 2021	139	173	125	173	2.2%	1.11 [0.99, 1.25]
Garg 2016	34	48	22	46	0.5%	1.48 [1.04, 2.11]
Liu 2018	215	239	178	237	2.9%	1.20 [1.10, 1.30]
Pillai 2018	30	56	15	48	0.3%	1.71 [1.06, 2.79]
**Subtotal (95% CI)**		**516**		**504**	**5.8%**	**1.21 [1.08, 1.34]**
Total events	418		340			
Heterogeneity: $Tau^2 = 0.00; Chi^2 = 4.94, df = 3 (P = 0.18); I^2 = 39\%$						
Test for overall effect: Z = 3.36 (P = 0.0008)						
**1.8.6 VDO stream from personal devices Vs Routine care**						
Archer 2021	219	254	205	259	3.0%	1.09 [1.01, 1.18]
Bearelly 2014	110	127	89	121	2.1%	1.18 [1.04, 1.34]
Jeon 2019	71	140	60	141	0.8%	1.19 [0.93, 1.53]
Jung 2022 (VDO personal devices vs Routine care)	96	104	40	53	1.5%	1.22 [1.04, 1.44]
Keswani 2020	412	424	403	406	4.1%	0.98 [0.96, 1.00]
Park 2016	229	250	198	252	3.1%	1.17 [1.08, 1.26]
Rice 2016	31	42	34	50	0.8%	1.09 [0.84, 1.41]
Walker 2022	103	111	94	102	3.0%	1.01 [0.93, 1.09]
**Subtotal (95% CI)**		**1452**		**1384**	**18.4%**	**1.09 [1.02, 1.18]**
Total events	1271		1123			
Heterogeneity: $Tau^2 = 0.01; Chi^2 = 40.40, df = 7 (P < 0.00001); I^2 = 83\%$						
Test for overall effect: Z = 2.37 (P = 0.02)						
**1.8.7 Web-based education Vs Routine care**						
Suzuki 2018	106	227	82	223	1.0%	1.27 [1.02, 1.58]
**Subtotal (95% CI)**		**227**		**223**	**1.0%**	**1.27 [1.02, 1.58]**
Total events	106		82			
Heterogeneity: Not applicable						
Test for overall effect: Z = 2.12 (P = 0.03)						
**Total (95% CI)**		**9441**		**8843**	**100.0%**	**1.10 [1.07, 1.13]**
Total events	8011		6846			
Heterogeneity: $Tau^2 = 0.00; Chi^2 = 180.68, df = 40 (P < 0.00001); I^2 = 78\%$						
Test for overall effect: Z = 7.12 (P < 0.00001)						
Test for subgroup differences: $Chi^2 = 14.76, df = 6 (P = 0.02), I^2 = 59.3\%$						

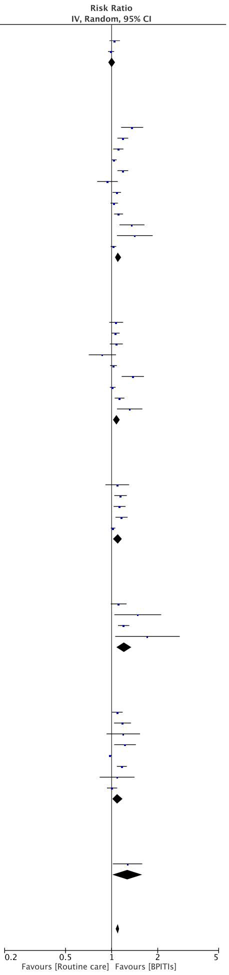

(B) quality of bowel preparation score

Study or Subgroup	BPITIs Mean	BPITIs SD	BPITIs Total	Routine care Mean	Routine care SD	Routine care Total	Weight	Std. Mean Difference IV, Random, 95% CI
**2.8.1 Computer-based education Vs Routine care**								
Veldhuijzen 2020	7.81	1.61	280	8	1.57	217	3.2%	−0.12 [−0.30, 0.06]
**Subtotal (95% CI)**			**280**			**217**	**3.2%**	**−0.12 [−0.30, 0.06]**
Heterogeneity: Not applicable								
Test for overall effect: Z = 1.32 (P = 0.19)								
**2.8.2 Mobile apps Vs Routine care**								
Kang 2016	−3.6	1.7	387	−4.5	1.8	383	3.3%	0.51 [0.37, 0.66]
Lam 2022	8.15	0.97	334	7.98	1.078	333	3.3%	0.17 [0.01, 0.32]
Lee 2016	7.7	1.1	71	7.24	0.8	71	2.5%	0.48 [0.14, 0.81]
Lorenzo-Zúñiga 2015	17.05	3.2	108	16.52	3.1	152	2.9%	0.17 [−0.08, 0.42]
Pan 2015	−3.6	1.7	387	−4.5	1.8	383	3.3%	0.51 [0.37, 0.66]
Sharara 2017	−6.4	1.95	80	−6.43	1.84	80	2.6%	0.02 [−0.29, 0.33]
van der Zander 2021	8.3	0.9	81	7.9	1.2	81	2.6%	0.38 [0.06, 0.69]
Walter 2021	7.6	1.57	246	6.7	1.56	243	3.2%	0.57 [0.39, 0.76]
Wang 2019 (Mobile apps vs Routine care)	6.81	1.1	127	5.78	1.33	62	2.6%	0.87 [0.55, 1.19]
Zhang 2018	7.1	1.2	542	6.3	1.4	534	3.4%	0.61 [0.49, 0.74]
**Subtotal (95% CI)**			**2363**			**2322**	**29.8%**	**0.43 [0.30, 0.57]**
Heterogeneity: $Tau^2 = 0.04; Chi^2 = 43.04, df = 9 (P < 0.00001); I^2 = 79\%$								
Test for overall effect: Z = 6.22 (P < 0.00001)								
**2.8.3 SMS Vs Routine care**								
Kim 2014 (SMS vs Routine care)	6.76	1.29	127	6.31	1.43	68	2.7%	0.33 [0.04, 0.63]
Lee 2015 (SMS vs Routine care)	6.8	1.3	126	6.3	1.4	67	2.7%	0.37 [0.07, 0.67]
Park 2015	−5	8.89	136	−5	5.93	135	2.9%	0.00 [−0.24, 0.24]
Patel 2018	8.67	0.74	60	8	1.48	95	2.5%	0.53 [0.21, 0.86]
Solonowicz 2022	7	0	329	7	0	274		Not estimable
Walter 2019	7.4	1.57	248	6.5	1.57	247	3.2%	0.57 [0.39, 0.75]
Wang 2019 (SMS vs Routine care)	6.44	1.1	128	5.78	1.33	62	2.6%	0.56 [0.25, 0.87]
**Subtotal (95% CI)**			**1154**			**948**	**16.6%**	**0.39 [0.20, 0.59]**
Heterogeneity: $Tau^2 = 0.04; Chi^2 = 16.23, df = 5 (P = 0.006); I^2 = 69\%$								
Test for overall effect: Z = 3.91 (P < 0.0001)								
**2.8.4 Telephone call Vs Routine care**								
Jung 2022 (Telephone vs Routine care)	7.3	1.1	101	6.6	1.1	53	2.5%	0.63 [0.29, 0.97]
Kim 2014 (Telephone vs Routine care)	7.09	1.15	126	6.31	1.43	68	2.7%	0.62 [0.32, 0.92]
Lee 2015 (Telephone vs Routine care)	7.1	1.2	125	6.3	1.4	67	2.6%	0.63 [0.32, 0.93]
Liu 2014	−3	2.3	262	−4.9	3.2	233	3.2%	0.69 [0.51, 0.87]
**Subtotal (95% CI)**			**614**			**421**	**11.0%**	**0.66 [0.53, 0.78]**
Heterogeneity: $Tau^2 = 0.00; Chi^2 = 0.23, df = 3 (P = 0.97); I^2 = 0\%$								
Test for overall effect: Z = 10.03 (P < 0.00001)								
**2.8.5 VDO stream from an on-site hospital device Vs Routine care**								
Bytzer 2007	−2.58	1.3	72	−2.33	0.74	90	2.6%	−0.24 [−0.55, 0.07]
Chen 2021	7.61	1.65	173	7.04	1.7	173	3.1%	0.34 [0.13, 0.55]
Liu 2018	−3.05	1.3	239	−4.18	1.4	237	3.2%	0.84 [0.65, 1.02]
Mi-Hee 2017 (VDO hospital vs Routine care)	−4	1.11	30	−5.17	1.96	14	1.3%	0.80 [0.15, 1.46]
Pillai 2018	−4.77	0	56	−6.85	0	48		Not estimable
**Subtotal (95% CI)**			**570**			**562**	**10.2%**	**0.41 [−0.08, 0.91]**
Heterogeneity: $Tau^2 = 0.22; Chi^2 = 37.13, df = 3 (P < 0.00001); I^2 = 92\%$								
Test for overall effect: Z = 1.66 (P = 0.10)								
**2.8.6 VDO stream from personal devices Vs Routine care**								
Back 2018	7.53	1.38	139	6.29	1.83	144	2.9%	0.76 [0.52, 1.00]
Bearelly 2014	6.99	1.87	127	6.43	2.54	121	2.9%	0.25 [0.00, 0.50]
Bowman 2014	8.01	0	179	7.41	0	171		Not estimable
Jeon 2019	−5.47	1.74	140	−5.97	1.78	141	3.0%	0.28 [0.05, 0.52]
Jung 2022 (VDO personal devices vs Routine care)	7.7	1.4	104	6.6	1.1	53	2.5%	0.84 [0.49, 1.18]
Keswani 2020	8.7	1	424	8.7	0.9	406	3.4%	0.00 [−0.14, 0.14]
Mi-Hee 2017 (VDO personal devices vs Routine care)	−4.37	1.63	29	−5.17	1.96	14	1.4%	0.45 [−0.19, 1.10]
Park 2016	−3.03	1.9	250	−4.21	1.9	252	3.2%	0.62 [0.44, 0.80]
Prakash 2013	−3	2.22	67	−5	2.96	66	2.4%	0.76 [0.41, 1.11]
Rice 2016	7	1.48	42	6.67	2.22	50	2.2%	0.17 [−0.24, 0.58]
Walker 2022	8	1.05	111	7.6	2.02	102	2.8%	0.25 [−0.02, 0.52]
Xu 2021	7.02	1.75	100	6.45	1.91	98	2.8%	0.31 [0.03, 0.59]
**Subtotal (95% CI)**			**1712**			**1618**	**29.3%**	**0.42 [0.23, 0.61]**
Heterogeneity: $Tau^2 = 0.08; Chi^2 = 60.52, df = 10 (P < 0.00001); I^2 = 83\%$								
Test for overall effect: Z = 4.26 (P < 0.0001)								
**Total (95% CI)**			**6693**			**6088**	**100.0%**	**0.42 [0.33, 0.52]**
Heterogeneity: $Tau^2 = 0.07; Chi^2 = 212.97, df = 35 (P < 0.00001); I^2 = 84\%$								
Test for overall effect: Z = 8.66 (P < 0.00001)								
Test for subgroup differences: $Chi^2 = 48.48, df = 5 (P < 0.00001), I^2 = 89.7\%$								

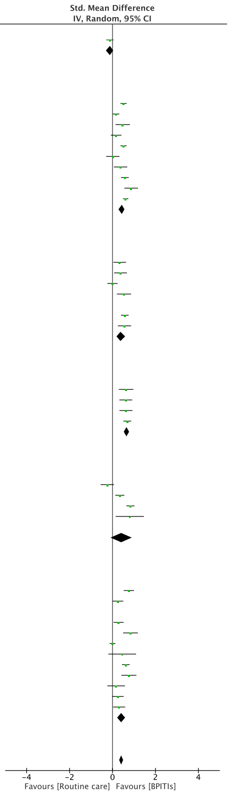

Supplementary S13: Forest plots of subgroup analyses of the effects of bowel preparation innovative technology instructions (BPITIs) on quality of bowel preparation score when compared with routine care by types of bowel preparation scale.

Study or Subgroup	BPITIs Mean	BPITIs SD	BPITIs Total	Routine care Mean	Routine care SD	Routine care Total	Weight	Mean Difference IV, Random, 95% CI
**2.14.2 Boston Bowel Preparation Scale (BBPS)**								
Back 2018	7.53	1.38	139	6.29	1.83	144	4.3%	1.24 [0.86, 1.62]
Bearelly 2014	6.99	1.87	127	6.43	2.54	121	3.5%	0.56 [0.00, 1.12]
Bowman 2014	8.01	0	179	7.41	0	171		Not estimable
Chen 2021	7.61	1.65	173	7.04	1.7	173	4.4%	0.57 [0.22, 0.92]
Jung 2022 (Telephone vs Routine care)	7.3	1.1	101	6.6	1.1	53	4.4%	0.70 [0.33, 1.07]
Jung 2022 (VDO personal devices vs Routine care)	7.7	1.4	104	6.6	1.1	53	4.2%	1.10 [0.70, 1.50]
Keswani 2020	8.7	1	424	8.7	0.9	406	5.3%	0.00 [−0.13, 0.13]
Kim 2014 (SMS vs Routine care)	6.76	1.29	127	6.31	1.43	68	4.2%	0.45 [0.04, 0.86]
Kim 2014 (Telephone vs Routine care)	7.09	1.15	126	6.31	1.43	68	4.2%	0.78 [0.39, 1.17]
Lam 2022	8.15	0.97	334	7.98	1.078	333	5.2%	0.17 [0.01, 0.33]
Lee 2015 (SMS vs Routine care)	6.8	1.3	126	6.3	1.4	67	4.2%	0.50 [0.10, 0.90]
Lee 2015 (Telephone vs Routine care)	7.1	1.2	125	6.3	1.4	67	4.2%	0.80 [0.40, 1.20]
Lee 2016	7.7	1.1	71	7.24	0.8	71	4.6%	0.46 [0.14, 0.78]
Patel 2018	8.67	0.74	60	8	1.48	95	4.4%	0.67 [0.32, 1.02]
Rice 2016	7	1.48	42	6.67	2.22	50	2.6%	0.33 [−0.43, 1.09]
Solonowicz 2022	7	0	329	7	0	274		Not estimable
van der Zander 2021	8.3	0.9	81	7.9	1.2	81	4.5%	0.40 [0.07, 0.73]
Veldhuijzen 2020	7.81	1.61	280	8	1.57	217	4.7%	−0.19 [−0.47, 0.09]
Walker 2022	8	1.05	111	7.6	2.02	102	4.0%	0.40 [−0.04, 0.84]
Walter 2019	7.4	1.57	248	6.5	1.57	247	4.8%	0.90 [0.62, 1.18]
Walter 2021	7.6	1.57	246	6.7	1.56	243	4.8%	0.90 [0.62, 1.18]
Wang 2019 (Mobile apps vs Routine care)	6.81	1.1	127	5.78	1.33	62	4.3%	1.03 [0.65, 1.41]
Wang 2019 (SMS vs Routine care)	6.44	1.1	128	5.78	1.33	62	4.3%	0.66 [0.28, 1.04]
Xu 2021	7.02	1.75	100	6.45	1.91	98	3.7%	0.57 [0.06, 1.08]
Zhang 2018	7.1	1.2	542	6.3	1.4	534	5.2%	0.80 [0.64, 0.96]
**Subtotal (95% CI)**			**4450**			**3860**	**100.0%**	**0.60 [0.42, 0.77]**
Heterogeneity: Tau² = 0.14; Chi² = 165.37, df = 22 (P < 0.00001); I² = 87%								
Test for overall effect: Z = 6.77 (P < 0.00001)								
**2.14.3 Harefield Cleansing Scale (HCS)**								
Lorenzo-Zúñiga 2015	17.05	3.2	108	16.52	3.1	152	100.0%	0.53 [−0.25, 1.31]
**Subtotal (95% CI)**			**108**			**152**	**100.0%**	**0.53 [−0.25, 1.31]**
Heterogeneity: Not applicable								
Test for overall effect: Z = 1.33 (P = 0.18)								
**2.14.4 Ottawa Bowel Preparation Scale (OBPS)**								
Jeon 2019	5.47	1.74	140	5.97	1.78	141	11.4%	−0.50 [−0.91, −0.09]
Kang 2016	3.6	1.7	387	4.5	1.8	383	13.7%	−0.90 [−1.15, −0.65]
Liu 2014	3	2.3	262	4.9	3.2	233	10.1%	−1.90 [−2.40, −1.40]
Liu 2018	3.05	1.3	239	4.18	1.4	237	13.8%	−1.13 [−1.37, −0.89]
Mi-Hee 2017 (VDO hospital vs Routine care)	4	1.11	30	5.17	1.96	14	4.3%	−1.17 [−2.27, −0.07]
Mi-Hee 2017 (VDO personal devices vs Routine care)	4.37	1.63	29	5.17	1.96	14	3.8%	−0.80 [−1.99, 0.39]
Pan 2015	3.6	1.7	387	4.5	1.8	383	13.7%	−0.90 [−1.15, −0.65]
Park 2015	5	8.89	136	5	5.93	135	1.9%	0.00 [−1.80, 1.80]
Park 2016	3.03	1.9	250	4.21	1.9	252	12.5%	−1.18 [−1.51, −0.85]
Pillai 2018	4.77	0	56	6.85	0	48		Not estimable
Prakash 2013	3	2.22	67	5	2.96	66	5.7%	−2.00 [−2.89, −1.11]
Sharara 2017	6.4	1.95	80	6.43	1.84	80	8.9%	−0.03 [−0.62, 0.56]
**Subtotal (95% CI)**			**2063**			**1986**	**100.0%**	**−1.00 [−1.27, −0.73]**
Heterogeneity: Tau² = 0.12; Chi² = 38.47, df = 10 (P < 0.0001); I² = 74%								
Test for overall effect: Z = 7.31 (P < 0.00001)								
**2.14.5 Not specified**								
Bytzer 2007	2.58	1.3	72	2.33	0.74	90	100.0%	0.25 [−0.09, 0.59]
**Subtotal (95% CI)**			**72**			**90**	**100.0%**	**0.25 [−0.09, 0.59]**
Heterogeneity: Not applicable								
Test for overall effect: Z = 1.45 (P = 0.15)								
Test for subgroup differences: Chi² = 97.50, df = 3 (P < 0.00001), I² = 96.9%								

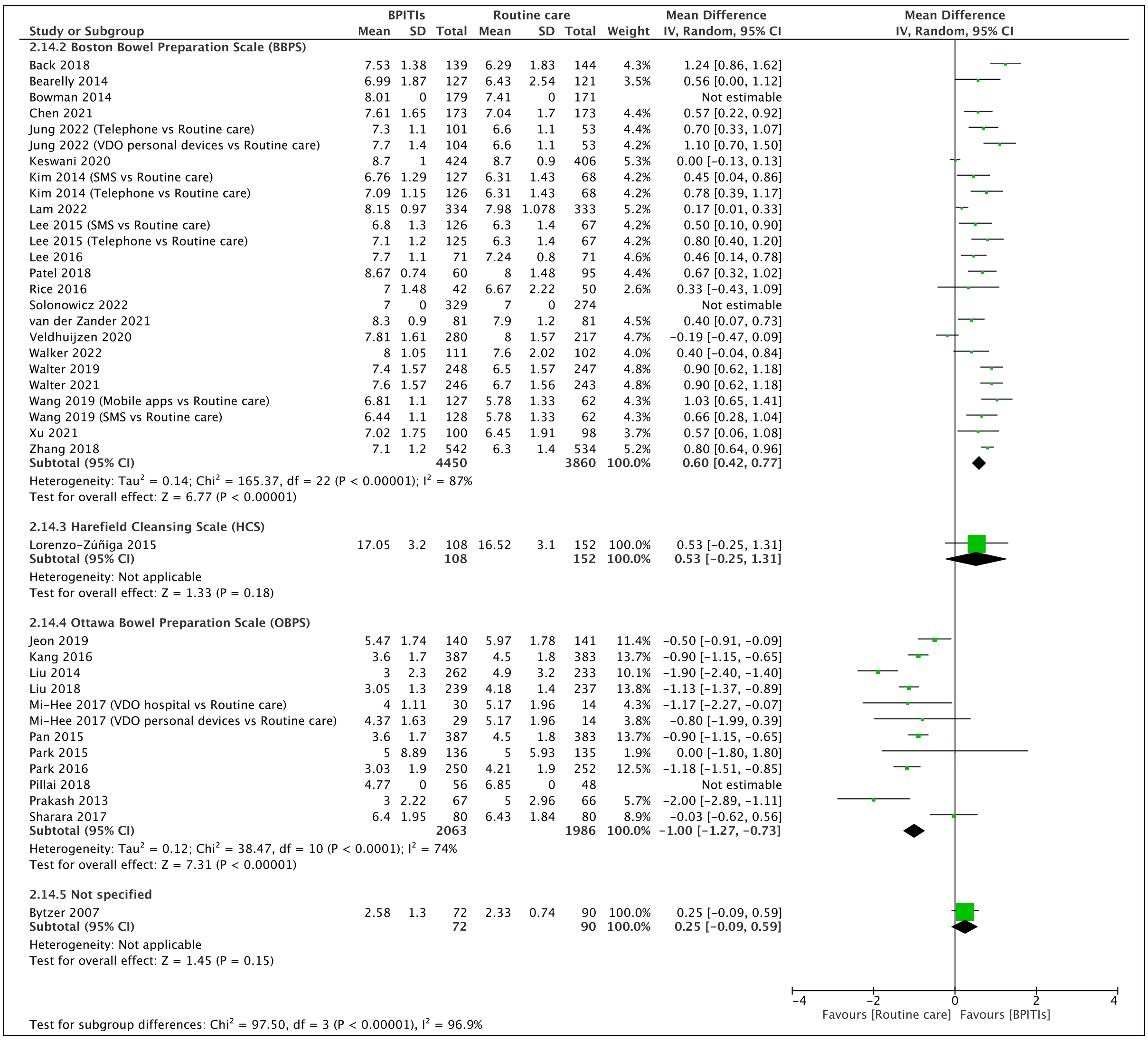

Supplementary S14: Forest plots of the effects of bowel preparation innovative technology instructions (BPITIs) on adequate bowel preparation adjusted for confounding factors and secondary outcomes when compared with routine care.

(A) Adequate bowel preparation (multivariable analysis)

Study or Subgroup	log[Odds Ratio]	SE	Weight	Odds Ratio IV, Random, 95% CI
Back 2018	2.1175	0.4993	13.3%	8.31 [3.12, 22.11]
Lam 2022	0.571	0.2145	25.1%	1.77 [1.16, 2.70]
Lee 2015 (SMS vs Routine care)	0.6523	0.5014	13.2%	1.92 [0.72, 5.13]
Lee 2015 (Telephone vs Routine care)	2.7967	1.0366	4.6%	16.39 [2.15, 125.01]
Park 2015	0.8454	0.2785	22.0%	2.33 [1.35, 4.02]
Park 2016	1.1282	0.2839	21.8%	3.09 [1.77, 5.39]
**Total (95% CI)**			**100.0%**	**2.92 [1.82, 4.67]**

Heterogeneity: $Tau^2 = 0.18$; $Chi^2 = 12.57$, $df = 5$ ($P = 0.03$); $I^2 = 60\%$
Test for overall effect: $Z = 4.46$ ($P < 0.00001$)

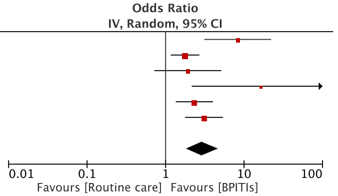

Study	Adjusted factors
Back, 2018	refrain from eating poorly digestible cereal grains
Lam, 2022	gender, hypertension, and diabetes
Lee, 2015	age, purgative ingested, preparation-to-colonoscopy time, and level of education
Park, 2015	compliance with diet instructions
Park, 2016	Sex and diabetes mellitus

(B) Polyp detection

Study or Subgroup	BPITIs Events	BPITIs Total	Routine care Events	Routine care Total	Weight	Risk Ratio IV, Random, 95% CI
Alvarez-Gonzalez 2020	130	303	117	302	7.0%	1.11 [0.91, 1.34]
Archer 2021	99	254	78	259	6.1%	1.29 [1.02, 1.65]
Bearelly 2014	34	64	15	36	3.2%	1.27 [0.81, 2.00]
Chen 2021	72	172	46	172	5.0%	1.57 [1.16, 2.12]
Garg 2016	23	48	16	46	2.9%	1.38 [0.84, 2.26]
Guo 2020	31	145	19	148	2.6%	1.67 [0.99, 2.81]
Jeon 2019	62	140	84	141	6.3%	0.74 [0.59, 0.94]
Jung 2022 (Telephone vs Routine care)	41	101	17	53	3.2%	1.27 [0.80, 2.00]
Jung 2022 (VDO personal devices vs Routine care)	32	104	17	53	2.9%	0.96 [0.59, 1.56]
Kang 2016	72	387	46	383	4.5%	1.55 [1.10, 2.18]
Kim 2014 (SMS vs Routine care)	57	127	22	68	3.8%	1.39 [0.94, 2.06]
Kim 2014 (Telephone vs Routine care)	61	126	22	68	3.9%	1.50 [1.02, 2.21]
Lee 2015 (SMS vs Routine care)	43	126	21	67	3.4%	1.09 [0.71, 1.67]
Lee 2015 (Telephone vs Routine care)	57	125	21	67	3.7%	1.45 [0.97, 2.18]
Lee 2016	23	71	15	71	2.4%	1.53 [0.87, 2.69]
Liu 2014	116	305	74	300	6.0%	1.54 [1.21, 1.97]
Liu 2018	32	239	32	237	3.2%	0.99 [0.63, 1.56]
Pan 2015	72	387	46	383	4.5%	1.55 [1.10, 2.18]
Park 2015	56	136	51	135	5.2%	1.09 [0.81, 1.46]
Park 2016	34	250	34	252	3.3%	1.01 [0.65, 1.57]
van der Zander 2021	44	87	36	86	4.7%	1.21 [0.87, 1.67]
Walker 2022	62	111	65	102	6.4%	0.88 [0.70, 1.09]
Wang 2019 (Mobile apps vs Routine care)	42	127	17	62	3.0%	1.21 [0.75, 1.94]
Wang 2019 (SMS vs Routine care)	39	128	17	62	2.9%	1.11 [0.69, 1.80]
**Total (95% CI)**		**4063**		**3553**	**100.0%**	**1.22 [1.10, 1.35]**
Total events	1334		928			

Heterogeneity: $Tau^2 = 0.03$; $Chi^2 = 44.66$, $df = 23$ ($P = 0.004$); $I^2 = 48\%$
Test for overall effect: $Z = 3.87$ ($P = 0.0001$)

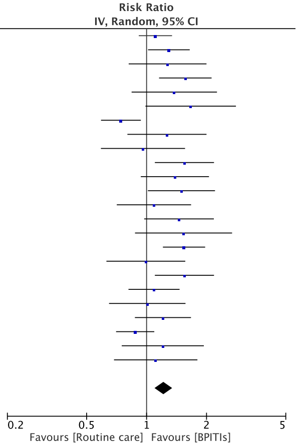

(C) Satisfied with intervention

Study or Subgroup	BPITIs Events	BPITIs Total	Routine care Events	Routine care Total	Weight	Risk Ratio IV, Random, 95% CI
Lee 2015 (SMS vs Routine care)	41	127	14	68	7.4%	1.57 [0.92, 2.67]
Lee 2015 (Telephone vs Routine care)	47	126	14	68	7.6%	1.81 [1.08, 3.04]
Mahmud 2019	21	21	0	0		Not estimable
Prakash 2013	50	67	45	66	16.6%	1.09 [0.88, 1.36]
Seoane 2020	195	382	97	373	17.3%	1.96 [1.61, 2.39]
Shaw 2001	41	44	32	42	17.7%	1.22 [1.01, 1.47]
Walter 2021	246	247	0	0		Not estimable
Wang 2019 (Mobile apps vs Routine care)	100	128	39	63	16.7%	1.26 [1.02, 1.56]
Wang 2019 (SMS vs Routine care)	102	129	39	63	16.7%	1.28 [1.03, 1.58]
**Total (95% CI)**		**1271**		**743**	**100.0%**	**1.38 [1.16, 1.65]**
Total events	843		280			

Heterogeneity: $Tau^2 = 0.04$; $Chi^2 = 20.65$, $df = 6$ ($P = 0.002$); $I^2 = 71\%$
Test for overall effect: $Z = 3.61$ ($P = 0.0003$)

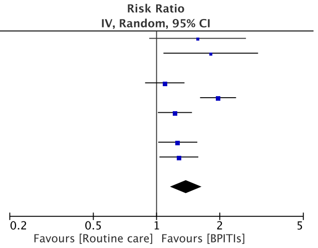

(D) Satisfaction score

Study or Subgroup	BPITIs Mean	BPITIs SD	BPITIs Total	Routine care Mean	Routine care SD	Routine care Total	Weight	Std. Mean Difference IV, Random, 95% CI
Back 2018	9.16	1.09	139	7.9	1.94	144	9.4%	0.79 [0.55, 1.04]
Chen 2021	8.68	1.7	173	8.16	2.15	173	9.6%	0.27 [0.06, 0.48]
Jung 2022 (Telephone vs Routine care)	9.5	1	97	8.2	2	51	8.5%	0.91 [0.55, 1.26]
Jung 2022 (VDO personal devices vs Routine care)	9.3	1.4	98	8.2	2	51	8.6%	0.67 [0.32, 1.02]
Lorenzo-Zúñiga 2015	8.7	1.6	108	6.9	2.7	152	9.3%	0.78 [0.52, 1.03]
Mi-Hee 2017 (VDO hospital vs Routine care)	41.66	5.24	30	33.93	5.8	14	5.6%	1.40 [0.70, 2.11]
Mi-Hee 2017 (VDO personal devices vs Routine care)	42.93	4.41	29	33.93	5.8	14	5.2%	1.81 [1.05, 2.56]
Seoane 2020	26.12	3.76	382	25.43	3.82	373	10.0%	0.18 [0.04, 0.32]
Sharara 2017	8.7	1.7	78	8.9	1.1	80	8.8%	-0.14 [-0.45, 0.17]
Shaw 2001	19.65	1.57	44	19	1.98	42	7.9%	0.36 [-0.06, 0.79]
Solonowicz 2022	4.56	0	899	4.59	0	825		Not estimable
van der Zander 2021	4.4	0.7	74	4.3	0.8	71	8.7%	0.13 [-0.19, 0.46]
Wen 2020	48.7	3.52	70	43.42	5.99	70	8.5%	1.07 [0.71, 1.42]
**Total (95% CI)**			**2221**			**2060**	**100.0%**	**0.62 [0.37, 0.86]**

Heterogeneity: $Tau^2 = 0.15$; $Chi^2 = 87.02$, $df = 11$ ($P < 0.00001$); $I^2 = 87\%$
Test for overall effect: $Z = 4.95$ ($P < 0.00001$)

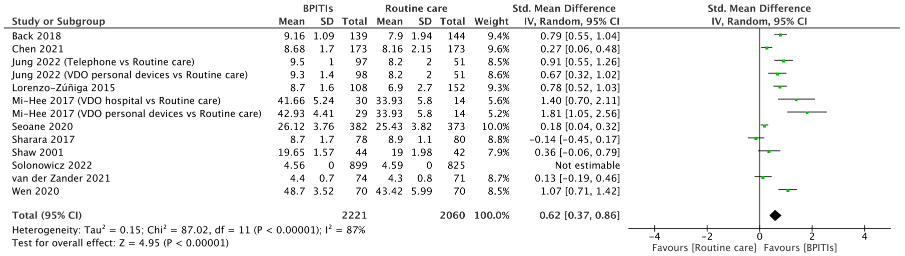

Supplementary S15: Funnel plots of original and sensitivity analyses for adequate bowel preparation and quality of bowel cleansing score outcomes.

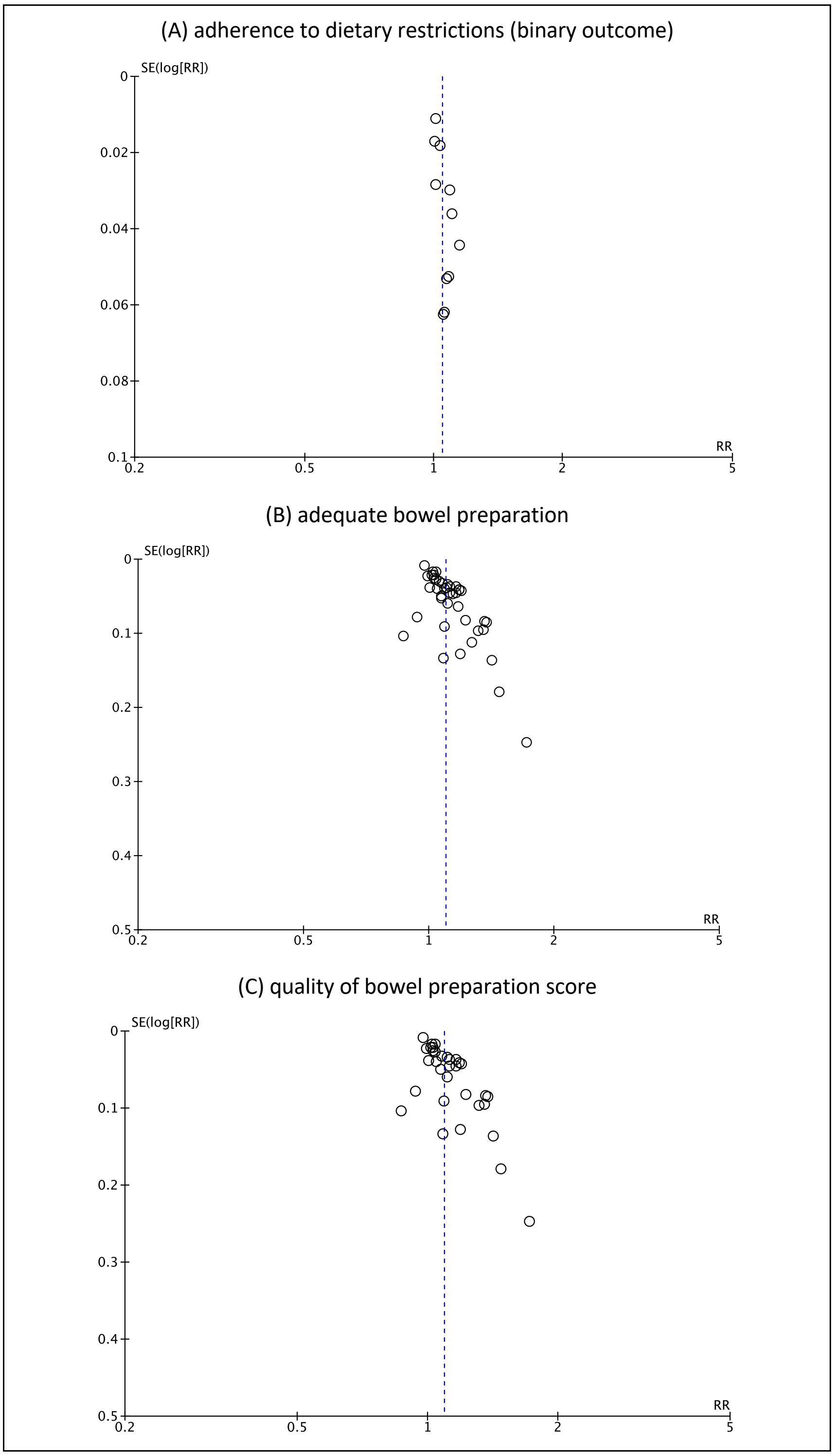

Supplementary S16: Summary of findings table for primary outcomes with reasons for grading the evidence.

Outcomes	Anticipated absolute effects* (95% CI)		Relative effect (95% CI)	№ of participants (studies)	Certainty of the evidence (GRADE)	Comments
	**Risk with Routine care**	**Risk with BPITIs**				
Adherence to overall instruction (binary outcome)	742 per 1,000	**891 per 1,000** (839 to 950)	**RR 1.20** (1.13 to 1.28)	3649 (8 RCTs)	⊕⊕⊕◯ Moderate [a]	
Adherence to purgative intake (binary outcome)	891 per 1,000	**936 per 1,000** (909 to 954)	**RR 1.05** (1.02 to 1.07)	3840 (9 RCTs)	⊕⊕⊕◯ Moderate [b]	
Adherence to dietary restrictions (binary outcome)	837 per 1,000	**921 per 1,000** (879 to 963)	**RR 1.10** (1.05 to 1.15)	4859 (10 RCTs)	⊕⊕⊕◯ Moderate [c]	
Adherence to overall instruction (continuous outcome)	The mean adherence with instructions (overall - continuous outcome) was **3.91** (maximum score = 6 scores)	MD **0.97 higher** (0.61 higher to 1.33 higher)	-	198 (1 RCT)	⊕⊕◯◯ Low [d]	
Adherence to purgative intake (continuous outcome)	-	SMD **0.8 SD higher** (0.39 higher to 1.21 higher)	-	370 (2 RCTs)	⊕◯◯◯ Very low [e,f,g]	
Adherence to dietary restrictions (continuous outcome)	-	SMD **0.99 SD higher** (0.35 higher to 1.63 higher)	-	370 (2 RCTs)	⊕◯◯◯ Very low [a,e,g]	
Adequate bowel preparation	789 per 1,000	**868 per 1,000** (845 to 892)	**RR 1.10** (1.07 to 1.13)	18284 (37 RCTs)	⊕⊕◯◯ Low [h,i]	
Quality of bowel preparation score	-	SMD **0.42 SD higher** (0.33 higher to 0.52 higher)	-	11724 (31 RCTs)	⊕⊕◯◯ Low [h,j]	

***The risk in the intervention group** (and its 95% confidence interval) is based on the assumed risk in the comparison group and the **relative effect** of the intervention (and its 95% CI).

CI: confidence interval; **MD:** mean difference; **RR:** risk ratio; **SMD:** standardised mean difference

GRADE Working Group grades of evidence
High certainty: we are very confident that the true effect lies close to that of the estimate of the effect.
Moderate certainty: we are moderately confident in the effect estimate: the true effect is likely to be close to the estimate of the effect, but there is a possibility that it is substantially different.
Low certainty: our confidence in the effect estimate is limited: the true effect may be substantially different from the estimate of the effect.
Very low certainty: we have very little confidence in the effect estimate: the true effect is likely to be substantially different from the estimate of effect.

Explanations

a. We downgraded one level for serious inconsistency due to substantial statistical heterogeneity (I2=76%)
b. We downgraded one level for serious inconsistency due to substantial statistical heterogeneity (I2=52%)
c. We downgraded one level for serious inconsistency due to substantial statistical heterogeneity (I2=73%)
d. We downgraded two levels for very serious imprecision due to only a single RCT with a limited sample size was available
e. We downgraded one level for serious risk of bias due to unclear information for selection bias
f. We downgraded one level for serious inconsistency due to substantial statistical heterogeneity (I2=49%)
g. We downgraded one level for serious imprecision
h. We downgraded one level for serious risk of bias due to some of the available studies were deemed to be at unclear or high risk of bias.
i. We downgraded one level for serious inconsistency due to substantial statistical heterogeneity (I2=78%)
j. We downgraded one level for serious inconsistency due to substantial statistical heterogeneity (I2=84%)
